# The application of machine learning to predict high-cost patients: A performance-comparison of different models using healthcare claims data

**DOI:** 10.1371/journal.pone.0279540

**Published:** 2023-01-18

**Authors:** Benedikt Langenberger, Timo Schulte, Oliver Groene

**Affiliations:** 1 Department of Health Care Management, Technische Universität Berlin, Berlin, Germany; 2 OptiMedis, Hamburg, Germany; 3 Department of Management & Innovation in Healthcare, Faculty of Health, University of Witten/Herdecke, Witten, Germany; Wroclaw University of Science and Technology, POLAND

## Abstract

Our aim was to predict future high-cost patients with machine learning using healthcare claims data. We applied a random forest (RF), a gradient boosting machine (GBM), an artificial neural network (ANN) and a logistic regression (LR) to predict high-cost patients in the following year. Therefore, we exploited routinely collected sickness funds claims and cost data of the years 2016, 2017 and 2018. Various specifications of each algorithm were trained and cross-validated on training data (n = 20,984) with claims and cost data from 2016 and outcomes from 2017. The best performing specifications of each algorithm were selected based on validation dataset performance. For performance comparison, selected models were applied to unforeseen data with features of the year 2017 and outcomes of the year 2018 (n = 21,146). The RF was the best performing algorithm measured by the area under the receiver operating curve (AUC) with a value of 0.883 (95% confidence interval (CI): 0.872–0.893) on test data, followed by the GBM (AUC = 0.878; 95% CI: 0.867–0.889). The ANN (AUC = 0.846; 95% CI: 0.834–0.857) and LR (AUC = 0.839; 95% CI: 0.826–0.852) were significantly outperformed by the GBM and the RF. All ML algorithms and the LR performed ´good´ (i.e. 0.9 > AUC ≥ 0.8). We were able to develop machine learning models that predict high-cost patients with ‘good’ performance facilitating routinely collected sickness fund claims and cost data. We found that tree-based models performed best and outperformed the ANN and LR.

## Introduction

The patterns of health service utilization as well as the resulting costs vary substantially across individuals within a given population. The fact that five percent of the population account for about half of the total populations healthcare costs is known to hold for various countries such as the US [1, 2], Germany [3, 4], Canada [5], Denmark [6], Japan [7], the Netherlands [8] or Australia [9]. The top five percent of patients among the cost distribution are referred to as high-cost patients (HCPs). Compared to non-HCPs, HCPs are more likely to have a low income, suffer from several chronic conditions [5], depend on multiple drugs [10], be of white skin color (at least in the US), have consulted a physician within the last year, be physically inactive and overweight, and have smoked within the past. The odds of becoming a HCP for individuals aged 80 or older within the next five years was found to be 37 times as high as for individuals aged younger than 30 [11].

The knowledge that only a small fraction of individuals accounts for a dramatic share of healthcare expenses leads to a question: How can the intensive resource consumption of HCPs be prevented to the benefit of health care systems, payers, and patients? An often-practiced approach to reduce cost is to focus on individuals which are already HCPs and try to reduce the future costs among those patients. However, a better approach could be to make accurate prognosis on which individuals will be HCPs in the future (e.g. next year) and intervene with appropriate measures preventively [12].

Variables such as age [7, 11, 13, 14], current high healthcare utilization/costs [7, 13, 15], hospitalization [13, 16], (number of) chronic conditions [13, 15], social deprivation [11, 13], patients general health status [11, 14], mental disorders [13, 14], obesity-related factors [7, 11] and diabetes or cardiovascular disease indicators [7] were found to be important predictors for becoming a HCP. Tamang et al (2017) [6] found that about one third of HCPs remain HCPs for the forthcoming year, while Wodchis et al (2016) [1] found that about 30 percent remain HCP for the following two years, once they are currently HCPs. Nevertheless, about two thirds of HCP were new to this group and labelled as ‘cost bloomers’ [6]. Among those, becoming a HCP is sometimes triggered by relatively rare or unforeseeable but expensive conditions [1], such as accidents. Consequentially, a certain percentage of HCP will most likely always stay unpredictable, even with sophisticated prediction tools. However, the remaining HCPs may be predictable. The accurate forecast of predictable future HCP based on observables is a goal with large cost-saving potential.

Previous studies that aimed to predict future HCPs relied on different methodological approaches. While some applied traditional approaches such as logistic regression (LR) [11–13, 15, 16], others applied machine learning (ML) methods [6, 14] or both [7, 17]. All of those studies reported the performance of the prediction models based on the area under the receiver operating curve (AUC)/c-statistic. The AUC is calculated as the area under the receiver operator curve. The receiver operator curve is a plot of sensitivity against false positive rate (1-specificity) of a given predictive tool using different decision thresholds for categorizing outcomes as either positive or negative [18]. AUC/c-statistic values are classified as fail (0.5–0.59), poor (0.6–0.69), fair (0.7–0.79), good (0.8–0.89) or excellent (0.9–1.0) [19]. Previous studies reached at worst fair [12] and at best excellent [16] performance. Noteworthy, prediction studies with different datasets cannot be compared reasonably [20]. However, the two studies that applied both ML and LR found that ML algorithms outperformed LR [7, 17]. Also in other applications, ML was found to (substantially) outperform LR [21–24]. This holds especially for the gradient boosting machine (GBM) and the random forest (RF) [24]. Apart from performance issues, ML yields practical advantages. Specifically, it is able to automatically detect non-linearities or interactions and may perform variable selection on its own, thus relying less on human input than LR [25–27]. This makes ML the preferred approach given the dataset used for this study. Therefore, in this study, LR was not defined as machine learning as follows previous research [28]. Nevertheless, LR was used as a reference model.

The objective of this paper was to assess and compare the performance of machine learning algorithms in predicting future high-cost patients (classification problem) using routinely collected healthcare claims and cost data from a German sickness fund.

## Methods

### Data source and study population

The data used for this study stems from a statutory health fund with insured individuals in the area of Hamburg, Germany. Data access for this research project was granted to the data processing organization (OptiMedis) based on a bilateral contract with the statutory health insurance company.

The dataset provided health related data over a period of three years, namely the years 2016 to 2018. The dataset with predictor variables of 2016 and outcomes of 2017 included observations of 20,984 individuals, while the dataset with predictor variables of 2017 and outcomes of 2018 consisted of 21,146 individuals. The dataset included information on demographics, care dependency, disease management program (DMP) participation, out- and inpatient diagnosis as well as data on the prescription of drugs for each insured individual. Further, the costs for each in- or outpatient treatment as well as for prescriptions were available. In the dataset obtained from OptiMedis, no missing values were present.

The diagnosis included in the dataset for in- and outpatient care are based on the ICD-10-GM (German modification). For the analysis in this paper, one- and three-digit level ICD-10-GM codes were included. The restriction to these levels was made as a trade-off between the number of variables, computation time and the precision of the diagnosis included. Consequentially, we included 243 variables reflecting diagnostic categories. We further added two variables indicating the number of different in- and outpatient diagnosis of an individual within a given year.

In addition, Anatomical Therapeutic Chemical (ATC) classification system groups for prescription drugs were included. For ATC codes, we focused on the three digits level for the purpose of building subgroups. This restriction led to 95 subgroups of pharmacological properties, also referred to as “therapeutic subgroup[s]” [29]. Furthermore, 14 “anatomical main group[s]” [29] were built out of the first digits. As in- and outpatient variables, prescription variables were included as dummies. A further variable indicating the absolute number of different ATC codes prescribed in the respective year was included. After elimination of variables that were either constant or yielded insufficient information, we finally utilized 653 predictor variables. In case an individual died within a year of feature determination, this individual was removed from the analysis as the cost within the next year would be zero. If an individual died within a year where the outcome was determined, it was included in the analysis.

### Outcome

For outcome definition, we followed previous research [7, 11–14, 16] and defined HCP as to be among the top five percent (i.e. above the 95 percent percentile) of the cost distribution based on all observed costs, namely costs for hospital care, outpatient care and prescription drugs. Together, these costs account for more than two third of the German statutory health insurance systems total spending [30].

### Performance indicators

Our main measure of performance was the AUC. Further, we report sensitivity, specificity, accuracy and the G-mean. Accuracy is defined as the number of true positives (TP) and true negatives (TN) divided by the sum of TP, TN, false positives, and false negatives [31]. The G-mean, also referred to as “geometric mean” [17], is the square root of the product of sensitivity and specificity [17]. Additionally, the area under the precision-recall curve was derived (AUC-PR). The AUC-PR acts as an important measure of robust predictive performance for imbalanced data, assessing the trade-off of sensitivity and the true positive rate [32].

### Statistical analysis

#### Machine learning methods

To predict future HCPs we applied three different machine learning algorithms, namely an artificial neural network (ANN) [33], a random forest (RF) [34] and a gradient boosting machine (GBM) [35].

An ANNs consists of multiple layers which consist of multiple units. The first layer is referred to as input layer, the last as output layer. Layers in between are called hidden layers. The ANN passes information through connections between the units (neurons) and layers [36]. When connections form cycles, the ANN is called recurrent ANN, while if connections are acyclic, it is called feed-forward ANN [37]. Each connection has a certain weight which represents the strength of the connection [36]. Weight values are derived by back-propagation [38]. Each neuron passes on an output called “activation” [33] to the next neuron(s), which then transform(s) it via an activation function (which can be of various forms) and forward(s) the information to the next neuron(s) in the system [33]. As a whole, the ANN is able to represent non-linear functions [36]. Certain parameters such as the activation function, the number of units within each layer and the number of layers can be tuned by the researcher [33]. For the purpose of this study, we applied a feed-forward ANN [38]. We varied the number of hidden layers [1, 2 or 3], learning rate [0.003, 0.005, 0.007], activation function [sigmoid (with/without dropout), maxout (with/without dropout), rectifier (with/without dropout)] and units [10, 20 or 100] within each layer. As measure of fit for weights, we applied the cross-entropy loss function [33, 38].

The RF is defined as a collection of classifiers with a tree structure, where the trees act as “independent identically distributed random vectors” [34]. Each tree gives a vote about which class the input belongs to (i.e., it classifies). Finally, the whole forest classifies the input as a consequence of the majority vote across it’s trees [34]. The depth of the trees can be specified. While increasing the trees depth reduces bias, increasing the number of trees reduces the forests variance, if the trees have low correlation. Low correlation is reached by choosing randomly selected variables for each tree grown, independently of the former tree [33]. For the RF applied in this paper, we varied the number of trees (500 or 1000) and the number of randomly selected features at each split (3, 5, 10 or √653). The maximum depth for each tree was set to 20.

As the RF, the GBM is also tree-based algorithm. The mathematical derivation consists of function estimation, numerical optimization, and the application of gradient boosting algorithm with steepest descent [35]. In contrast to RF, the GBM’s trees are not independent of each other. Each tree derived in each step depends on the models fit from the former steps [33]. We varied the number of trees (100, 250, 500 or 1000) and the maximal depth (1 or 2) as tuning parameters in our application.

Previously mentioned hyperparameters were tuned for all models. Hyperparameter optimization for each method was performed for each model using grid search [39, 40]. Final hyperparameters selected for each model are shown in S2 Table.

As in comparable studies [7, 17], a logistic regression was used as baseline comparison model. Statistical difference between the AUC values of different models was assessed, where relevant, using the method of Delong et al 1988 [41].

#### Training data, test data and cross-validation

To train, validate and test our models, we followed common procedures and split our dataset into training (75%) and test (25%) data. Therefore, we followed Moturo et al. [17] and Osawa et al. [7] and used a training dataset with variables from a year t_0_ and HCP status data (= outcome) from a following year t_1_, so that the models predicted outcomes of t_1_ with data from t_0_. On this training dataset, 5-fold cross-validation (CV) was performed. In general, *k*-fold CV is a procedure in which the dataset is split into k parts. Then, *k*-1 parts are used for training, and the part not used for training is used for validation. This is done *k* times, until each part of the dataset is used once for validation and *k*-1 for training. Based on models’ performance during CV, we performed model selection [33]. That is, we applied CV to all algorithms (RF, ANN, GBM) with different hyperparameter specifications and chose the best performing hyperparameter specification for each algorithm. Nevertheless, since we selected models based on their performance with CV on training data, we could not rule out that our models overfit with respect to the training data [33]. Therefore, we applied the selected model of each algorithm to an independent unforeseen test dataset for performance assessment. Before being applied, the selected models were trained again on the full training dataset. The test dataset was constructed of variables from t_1_ and outcomes of t_2_. Within this study, t_0_, t_1_ and t_2_ correspond to the years 2016, 2017 and 2018, respectively.

#### Analysis

To run the desired model in the statistical software R, version 4.0.5, the package H2O (cluster version 3.36.0.4) from the company H2O.ai was used (see [42] for further information).

To identify the most important variables among the 653 predictors, we performed a variable importance analysis for each algorithm. Standardized methods for calculating variable importance are included in the h2o package in R. For the ANN, the h2o package exploits the approach by Gedeon [43, 44]. This method sums the magnitude of the weights of the neurons’ connections for each input variable and derives its relative importance. For the GBM and the RF, the h2o package calculates the variable importance as the change in squared error induced by the inclusion of the respective variable [45]. Additionally, for the best performing model, we conducted a SHapley Additive exPlanations (SHAP) analysis [46] to explore how the most important variables influenced classification based on the test dataset. SHAP analysis, an approach with origins from game-theory, illustrates how each variables value influenced the respective ML models predicted class probability for each individual observed. It additionally ranks variables with respect to their influence on predicted class probability for the whole sample [47, 48]. Due to different approaches of deriving the variables importance, SHAP analysis derived variable importance may differ from the variable importance derived by squared error reduction.

95% asymptotic confidence intervals for the all performance measures except the AUC were calculated by the formula: confidence interval = performance measure ± 1.96 * standard error(performance measure), where the standard error (SE) is: SE = square root[performance measure * (1—performance measure) / n] [49]. For AUC, the confidence intervals were derived using the method of LeDell et al 2015 [50].

## Results

### Data extraction

To create training and validation data, a dataset based on observations of 653 health claims and demographic variables from 20,984 individuals of the year 2016 and the individuals respective cost data from the year 2017 was selected. This dataset was then split randomly in 75 percent training data and 25 percent validation data. The training dataset contained 15,738 insured individuals, while the validation set contained 5,246 insured individuals, respectively. The test dataset contained observations of 21,146 individuals and consisted of healthcare claims and demographics data from the year 2017 and cost data from the year 2018.

### Summary statistics

HCPs accounted for about 46.8 percent of the total costs (Table 1). They had a longer average duration of care dependency, a substantially higher age (about 20 years on average), a substantially higher share of individuals older than 65 and spent more time in a DMP, on average. Further, the average costs of a HCPs were about 24,983 € per year, while, for non-HCPs, the average costs are only 1,496 € per year. Besides that, HCPs showed higher values for variables indicating multimorbidity such as the number of different hospital or outpatient diagnostic categories as main diagnosis as well as different ATC prescription subcategories. Note that all differences between the two groups means were statistically significant (column 3 of Table 1).

**Table 1:** Summary statistics of HCPs and non-HCPs, based on a subset of variables and the 2016/2017 dataset.

Variables	Non-HCPs (n = 19,935)	HCPs (n = 1,049)	p-value
**Duration of care dependency (years)**	0.2	0.9	< 2.2e-16
**Age**	40.7	60.4	< 2.2e-16
**Age ≥ 65 (%)**	19.1	50.0	< 2.2e-16
**Fraction who are male (%)**	47.0	51.1	< 2.2e-16
**Duration of DMP (years)**	1.3	3.6	< 2.2e-16
**Average Costs per year in €**	1,496	24,983^a^	< 2.2e-16
**Number of different hospital main diagnosis**	0.2	2.3	< 2.2e-16
**Number of different ATC prescriptions**	3.2	5.7	< 2.2e-16
**Number of different outpatient main diagnosis**	6.5	9.4	< 2.2e-16
**Share of total health care expenditures (%)**	53.2	46.8	-

^a^Among HCPs, high-cost males had average costs of 27,292.23 €, while female HCPs had average costs of 22,574 €. p-values were derived with Welch two sample t-test for continuous variables or two proportions test for comparing proportions.

### Model selection

As described, we varied various tuning parameters for each algorithm to perform model selection. The selected models are presented in Table 2. We found the GBM with a maximal depth of two and a number of 500 trees performed best. For the random forest, the model with the number of variables randomly selected at each node set to the square root of the number of all available variables as well as with 500 trees worked best. Finally, the best performing ANN had two hidden layers, 10 units per hidden layer, the maxout activation function with dropout and a learning rate of 0.003 (see also S2 Table).

**Table 2:** Algorithm performance on cross-validated training data (n = 20,984).

	Performance measure				
Model	AUC	Sensitivity	Specificity	Accuracy	G-mean
**Logistic regression**	0.842 (0.829–0.854)	0.763 (0.758–0.769)	0.777 (0.772–0.783)	0.777 (0.771–0.782)	0.770 (0.765–0.776)
**Gradient boosting machine**	0.881 (0.871–0.892)	0.845 (0.840–0.849)	0.767 (0.761–0.772)	0.771 (0.765–0.777)	0.805 (0.799–0.810)
**Random forest**	0.882 (0.871–0.892)	0.829 (0.824–0.834)	0.773 (0.767–0.778)	0.776 (0.770–0.781)	0.800 (0.795–0.806)
**Artificial neural network**	0.842 (0.830–0.853)	0.800 (0.794–0.805)	0.745 (0.739–0.751)	0.748 (0.742–0.754)	0.722 (0.766–0.778)

95% confidence intervals in parenthesis.

On validation data, RF and the GBM significantly outperformed LR and the ANN (see non-overlapping confidence intervals in Table 2). The RF reached the highest AUC with a value of 0.882. LR and ANN performed equally good (Table 2).

### Performance evaluation

To evaluate algorithm performance, we applied our selected models to unforeseen test data of the subsequent year. Therefore, models selected based on the validation dataset performance were trained with the full dataset of 2016/2017 as described in the methods section, and then applied to the test dataset of 2017/2018.

**Fig 1:**
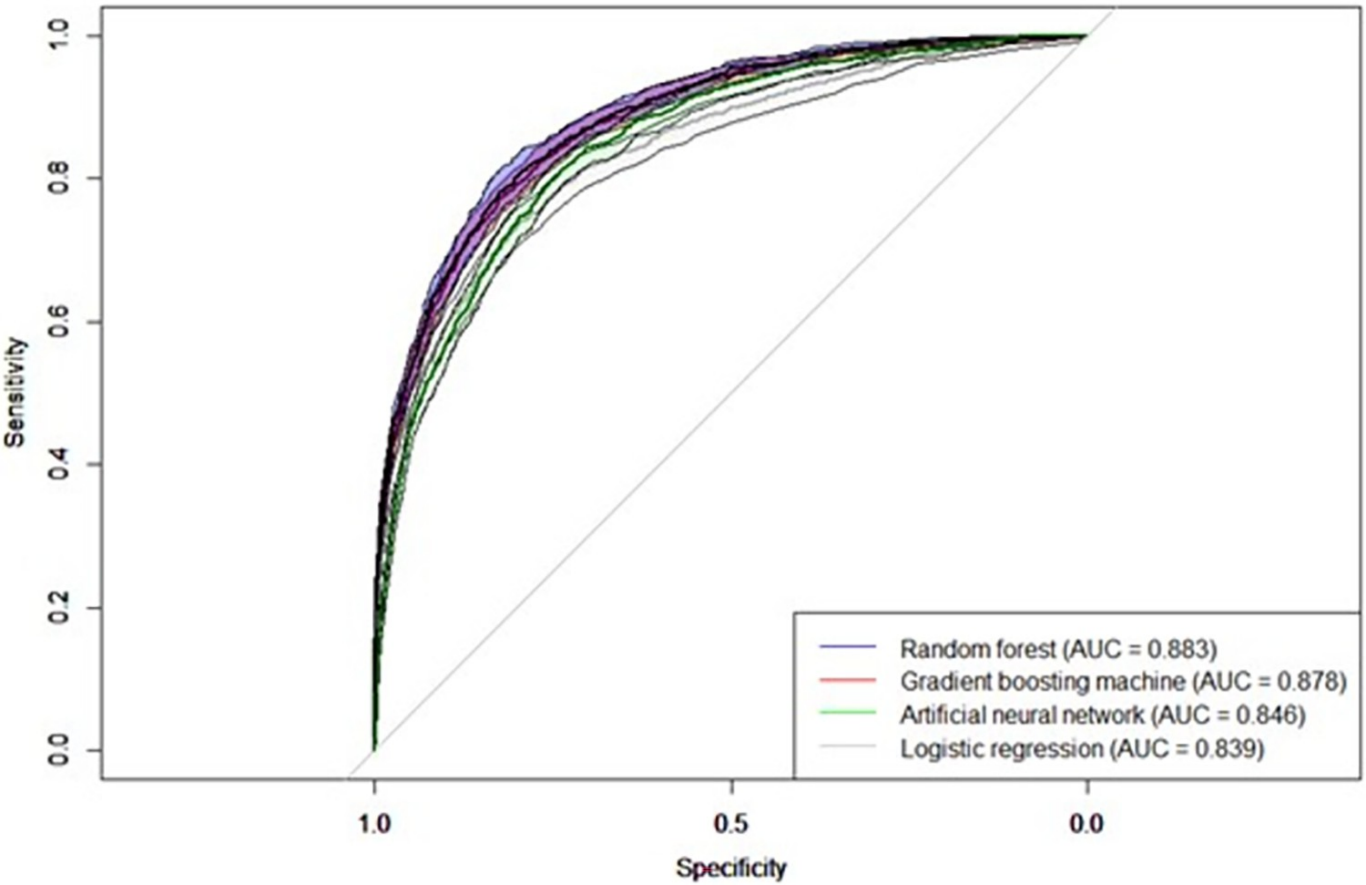
Receiver operating curves of models’ performance on test data, including confidence intervals for 95% confidence level. Confidence intervals were calculated using the cvAUC package in R.

As on training data, RF remained the best performing model on test data (Table 3), however not statistically significantly different from the GBM (p-value = 0.076 derived by the method of Delong et al 1988 [41]). Further, both GBM and RF outperformed the ANN and LR statistically significantly (p-values: GBM vs. ANN = 0.000; GBM vs. LR = 0.000; RF vs. ANN = 0.000; RF vs. LR = 0.000) (see Fig 1 for AUC plots).

**Table 3:** Selected models performance on unforeseen test data of 2017/2018 (n = 21,146).

	Performance measure				
Model	AUC	Sensitivity	Specificity	Accuracy	G-mean
**Logistic regression**	0.839 (0.826–0.852)	0.740 (0.734–0.746)	0.793 (0.787–0.798)	0.790 (0.796–0.785)	0.766 (0.760–0.772)
**Random forest**	0.883 (0.872–0.893)	0.780 (0.774–0.786)	0.823 (0.817–0.828)	0.820 (0.815–0.825)	0.801 (0.796–0.825)
**Gradient boosting machine**	0.878 (0.867–0.889)	0.770 (0.765–0.776)	0.824 (0.819–0.829)	0.821 (0.816–0.826)	0.797 (0.791–0.826)
**Artificial neural network**	0.846 (0.834–0.857)	0.773 (0.767–0.779)	0.750 (0.755–0.761)	0.756 (0.751–0.762)	0.764 (0.758–0.770)

95% confidence intervals in parenthesis.

**Fig 2:**
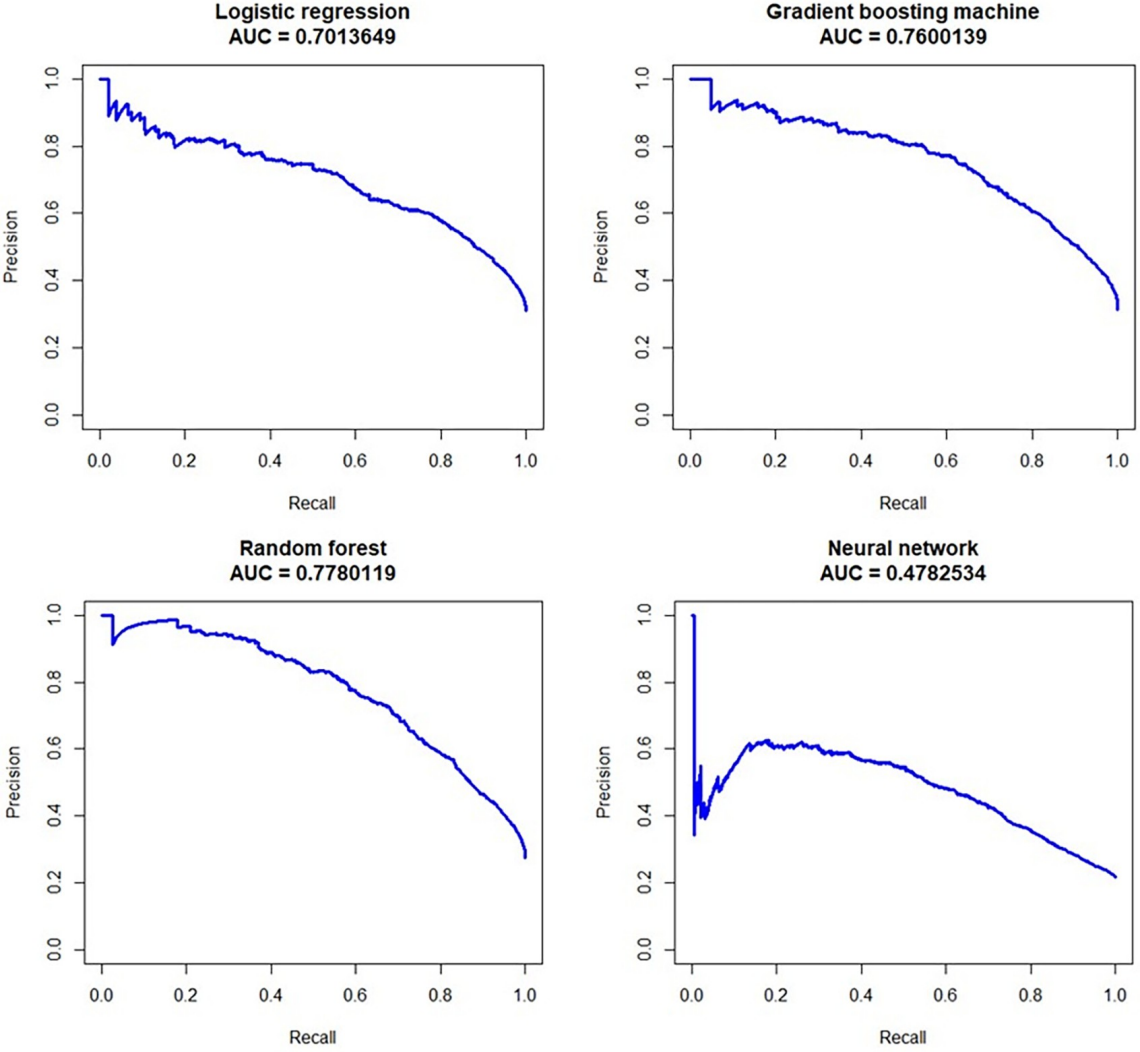
Precision-recall curves of model’s performance on test data.

The final tuning parameter values for all models can be found in S2 Table. We also show the confusion matrix of the predictions at the sensitivity-specificity trade-off that maximized the g-mean (S3 Table). Precision-recall curves were plotted and the area under the precision-recall curve derived in Fig 2. It could be observed that performance for all models decreased, compared to the AUC. Performance decline for RF, GBM and LR was slightly above 0.1. Predictive ability remained reasonable. However, the NN’s performance decreased dramatically to only 0.43 on the AUC-PR, implying that the NN was not able to reasonably deal with the imbalanced data when trading-off sensitivity and the true positive rate. We tested the “balance_class” parameter in the h2o.deeplearning() method and found that this did not improve results to a noteworthy extend.

### Variable importance

Variable importance was calculated as described in the ‘*Analysis’* section. For simplicity, we only report the top five predictors for each algorithm.

**Fig 3:**
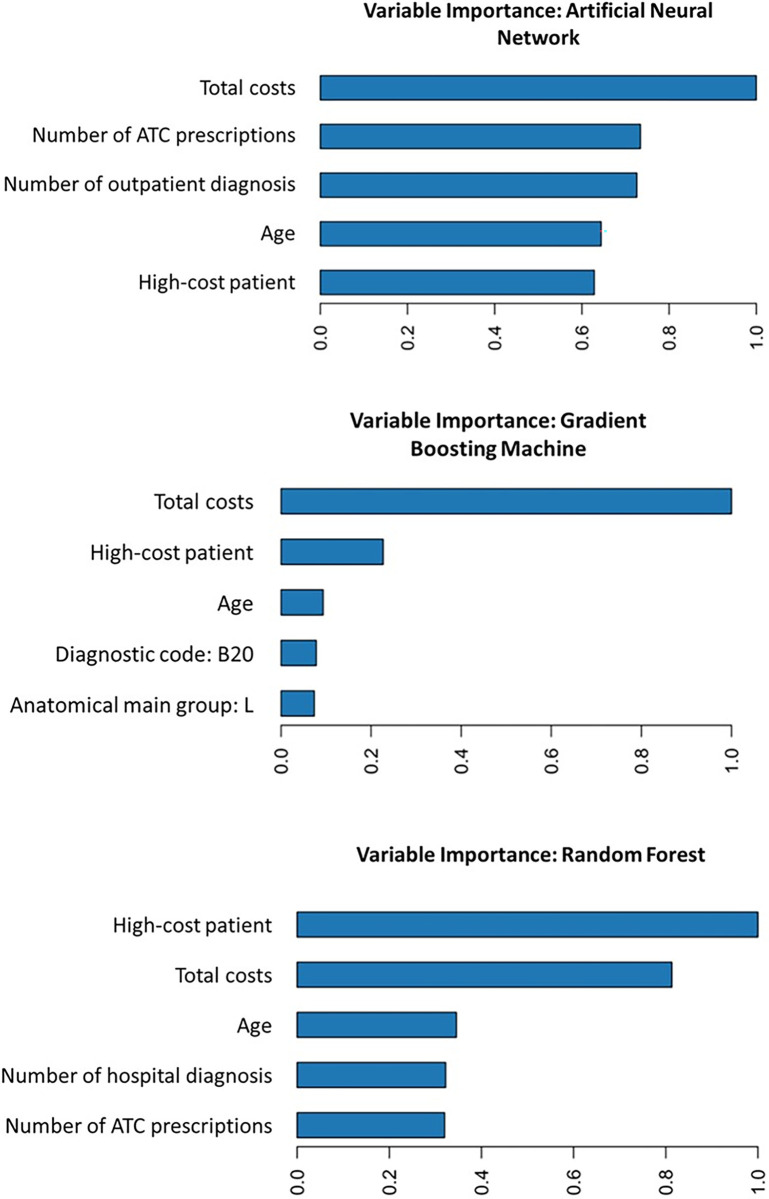
Variable importance for each selected model/algorithm. a) Artificial neural network, b) Gradient boosting machine, c) Random forest.

Fig 3 shows the five most important variables identified by each algorithm. The most important predictor is set to 1 (see x-axis), while the other four top five predictors are ranked with their importance relative to the most important predictor (see their x-axis values). All algorithms ranked patients age, HCP dummy of the current year and total costs of the current year among the top five most important predictors for predicting next year’s HCPs. The ANN also ranked the number of outpatient diagnosis and the number of ATC codes as most important predictors. The GBM ranked one diagnostic code (B.20 - the code for an infectious disease as a consequence of a HIV-disease) [51] and the anatomical main group L for antineoplastic and immunomodulating agents [52] among the top predictor variables.

SHAP analysis revealed further insights into how variables influenced predicted class probabilities for the best performing model (RF).

**Fig 4:**
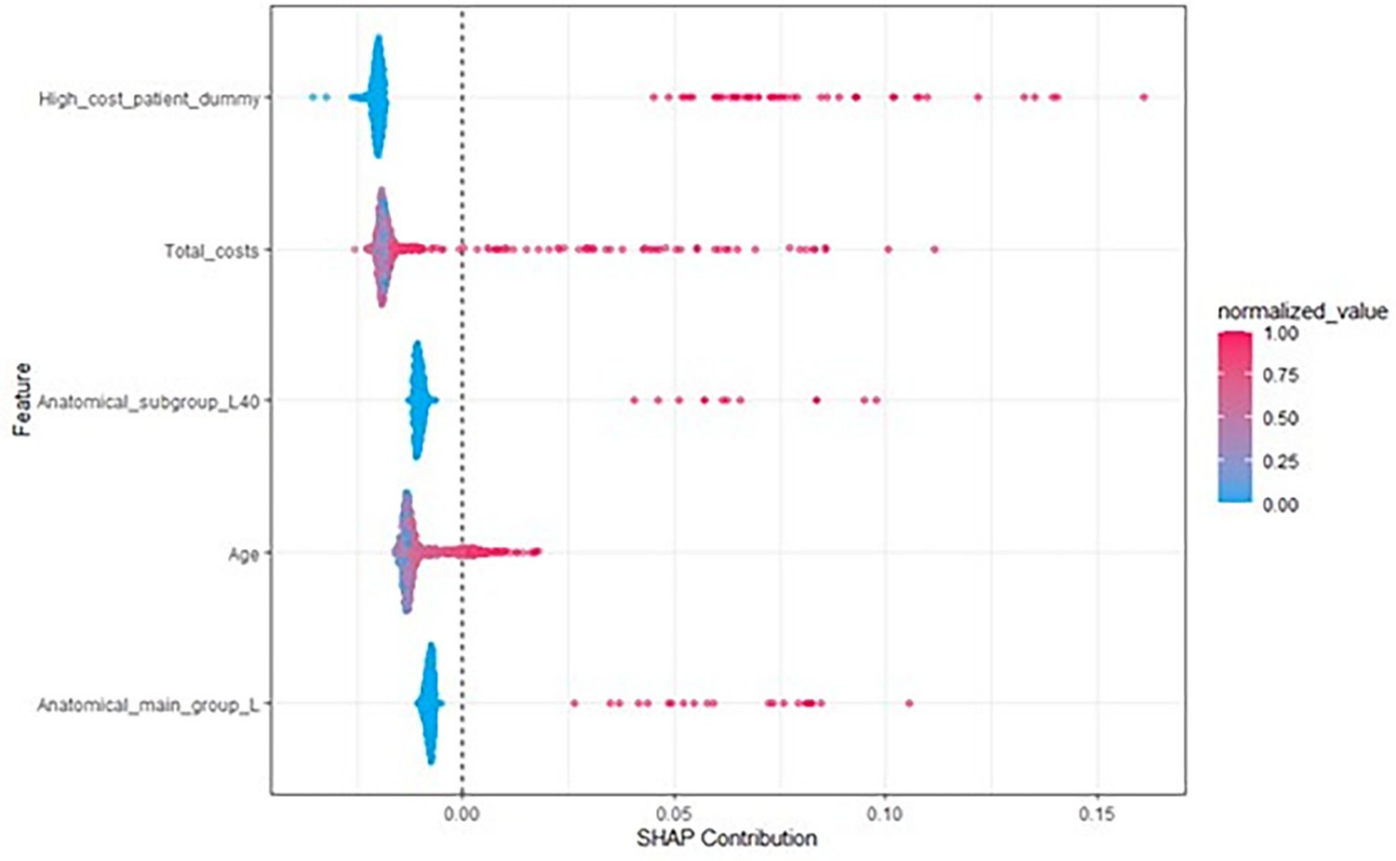
SHAP analysis for the random forest on test data. Each dot represents an individual.

One can observe that the ranking of the variables in the SHAP analysis differs from the one of the previously shown variable importance plots. This is due to the different calculation methods (squared error reduction vs. influence on predicted probabilities, see ‘Analysis’). Each dot in Fig 4 represents an individual. A blue dot indicates a small value of the respective variable, while a red dot indicates a high value. The x-axis represents the influence of the variable on the predicted class probability. Thus, we see that having been a HCP, high costs, the intake of medications from the ATC therapeutic subgroup ‘L40’ (immunosuppressants), the intake of medications of the ATC anatomical main group ‘L’ (antineoplastic and immunomodulating agents) and high age in the last year increase the likelihood of becoming a HCP within the next year.

## Discussion

In line with various previous studies [1–3, 5–7, 11], we found that HCPs, defined as top five percent among the cost distribution, accounted for almost half of healthcare costs in a dataset from a large German sickness fund.

For predictions with ML algorithms, we relied on sickness fund claims data which comes without extra costs for sickness funds to be collected. Other authors with the aim to predict HCPs exploited additional variables such as clinical data [7, 11] or behavioral data [11], or relied solely on different data such as text from patient records [12]. Further survey data was often used [11, 14, 15], which is not as continuously available as sickness fund data, jeopardizing the aim of making continuous up-to-date predictions for the underlying population. While including additional information such as clinical, socioeconomic, or behavioral (survey) data may be beneficial, in practice it is more difficult to be collected on a large scale, up-to-date and at low costs.

To our knowledge, our developed models outperformed all other models from previous studies that aimed to predict HCPs based on AUC (see S4 Table). Our tree-based models reached ‘good’ performance based on AUC. Only one other study [16] reported a higher AUC while applying a LR. However, this study [16] exploited hospital claims, not sickness fund data, thus relying only on patients already receiving medical treatment associated with inpatient care. This makes their setting and data hard to compare with the setting and data considered in this study. Comparing our results to other studies with a comparable study population, we argue that the high performance of our models is noteworthy since we did not include additional information on top of routinely collected health insurance data, such as others did [7, 11]. This finding shows that routinely collected health claims and demographic sickness fund data are sufficient to establish high-performing prediction models. We also found that logistic regression was statistically significantly outperformed by tree-based ML algorithms, in line with previous research [7, 17, 24, 53–55] and to our knowledge a new contribution to the research question at hand. For imbalanced datasets such as ours, balancing the datasets might improve model performance [56]. E.g. Moturo et al. [17] found that balancing classes improves model performance. In contrast, we did not find that balancing the training data did improve predictive performance of our algorithms, even though we tested this approach by using the balance_classes command in the h2o package. This command balances the classes either by under- or oversampling [57]). It is also of relevance that despite model performance issues, the AUC–which was our main outcome metric–is not negatively affected by imbalanced datasets [58]. In addition to the AUC, we plotted precision-recall curves to assess the trade-off of sensitivity and the true-positive rate among decision thresholds. Tree-based models performed best on this metric, showing reasonable performance albeit the highly skewed data with respect to the small minority class. Further, we found that the model selection procedure with a validation dataset was able to identify models which also performed best on the test data or did not differ statistically significant in their performance from the best model based on test data. Nevertheless, we do not know how our models perform on datasets from other sickness funds (i.e. other populations). Depending on the dataset size and the populations characteristics, other algorithms and model specifications than found in this application may turn out to perform best. Therefore, we recommend repeating the process of training and validation including model selection applications on other datasets. If feasible, a comparison to the models identified through model selection in this paper might be performed to see how the same algorithm specifications perform on different data.

The findings of our study highlight that ML approaches can identify future HCPs with reasonable performance using only routinely available data, thus opening the possibility to reach this population with specific measures. If effective measures that prevent potential HCPs with a high probability from actually becoming HCPs can be implemented, substantial expenditures can be prevented at healthcare system level. This facilitates a shift from healthcare system focusing on sickness towards a healthcare system focusing on prevention and proactive case- and care-management [59].

Most important predictors found in this study were in line with previous evidence. Current healthcare costs [7] and age [7, 11, 13, 14] were also most important predictors in previous research. The fact that a substantial amount of HCP patients remain so for the following year [1, 6] may explain why the HCP indicator for the current year has been ranked as highly important predictor for becoming a HCP in the following year. As SHAP analysis revealed, high costs/being a HCP in the current year is associated with an increased probability of being HCP in the next year, as was higher age. Adding to previous evidence, we found that the number of in- or outpatient diagnosis as well as the number of ATC prescriptions may act as top predictors for identifying future HCPs. These variables are easy to create in sickness fund data and therefore provide potential to improve predictive performance with low effort.

Nevertheless, the study comes with several limitations. First, we did only include variables of one year to predict future HCPs. Other authors used data of more than one year, even though they found that adding data of further years did not improve performance notably [7]. Second, certain variables identified to be highly important predictors by algorithms are either unchangeable (age) or indicate patients already have high healthcare utilization (total costs/current HCP), thus limiting potential for preventive measures. Third, we did only exploit structured, routinely collected healthcare claims data available to sickness funds. There are also other, more unstructured sources of data permanently produced in the healthcare sector, e.g., discharge letters. If medical text data such as discharge letters were included, predictive performance might have further improved. Former research already indicated that models facilitating text data are able to make predictions about HCP status with reasonable performance [12]. Fourth, ML is a fast evolving and dynamic research field. New algorithms are developed on a rapid pace (see e.g. [60, 61] for recently developed methods). Further research may therefore apply newly developed ML algorithms to comparable datasets and test whether they are able to outperform well-established ones such as those applied in this paper. Further research is also needed to explore whether adding more unstructured but routinely produced data sources such as text data might yield additional benefits when used on top of claims data. In general, adding other data sources (structured or unstructured) may shed a light on how and which further data sources improve performance on top of sickness fund claims data.

Future research is required to develop effective measures for patients which are predicted to be future HCPs. Additional variables which can be created out of health insurance datasets might be subject to future research. Once implemented in practice, algorithms need continuous training to catch up with medical innovation, which continuously changes costs and healthcare outcomes for various indications.

## Conclusion

We were able to apply common ML algorithms to predict future HCPs with ‘good’ performance, exploiting routinely collected sickness fund data. Some important predictors were in line with previous evidence, while we further found that the number of in- or outpatient diagnosis and drug prescriptions are highly relevant predictors. Apart from ‘good’ predictive models, preventive measures will be necessary in order to derive benefits from accurate predictions.

## Supporting information

S1 Table Overview of all used input variables in the training/validation (2016) test (2017) dataset. (DOCX)

S2 Table Selected hyperparameters for all machine learning algorithms after grid search on validation data. (DOCX)

S3 Table Confusion matrix of the random forest. (DOCX)

S4 Table Comparison with other studies. (DOCX)

## References

[1] Wodchis WP, Austin PC, Henry DA. A 3-year study of high-cost users of health care. CMAJ. 2016; 188:182–8. doi: 10.1503/cmaj.150064 . 26755672 PMC4754179

[2] Cohen SB. The concentration of health care expenditures in the U.S. and predictions of future spending. JEM. 2016; 41:167–89. doi: 10.3233/JEM-160427

[3] Busse R. Wettbewerb im Gesundheitswesen–eine Gesundheitssystemperspektive. Zeitschrift für Evidenz, Fortbildung und Qualität im Gesundheitswesen. 2009; 103:608–15. doi: 10.1016/j.zefq.2009.10.011 20120187

[4] Lange L, Pimperl A, Schulte T, Groene O, Tanke M. Hochkostenversicherte in Deutschland: Leistungs- und Kostenprofile. Z Evid Fortbild Qual Gesundhwes. 2020; 153–154:76–83. Epub 2020/06/12. doi: 10.1016/j.zefq.2020.05.007 . 32540309

[5] Rosella LC, Fitzpatrick T, Wodchis WP, Calzavara A, Manson H, Goel V. High-cost health care users in Ontario, Canada: demographic, socio-economic, and health status characteristics. BMC Health Serv Res. 2014; 14:1–13. doi: 10.1186/s12913-014-0532-2 . 25359294 PMC4221677

[6] Tamang S, Milstein A, Sørensen HT, Pedersen L, Mackey L, Betterton J-R, et al. Predicting patient ’cost blooms’ in Denmark: a longitudinal population-based study. BMJ Open. 2017; 7:e011580. doi: 10.1136/bmjopen-2016-011580 . 28077408 PMC5253526

[7] Osawa I, Goto T, Yamamoto Y, Tsugawa Y. Machine-learning-based prediction models for high-need high-cost patients using nationwide clinical and claims data. NPJ Digit Med. 2020; 3:148. Epub 2020/11/11. doi: 10.1038/s41746-020-00354-8 . 33299137 PMC7658979

[8] Bakx P, O’Donnell O, van Doorslaer E. Spending on Health Care in the Netherlands: Not Going So Dutch. Fiscal Studies. 2016; 37:593–625. doi: 10.1111/j.1475-5890.2016.12114

[9] Calver J, Brameld KJ, Preen DB, Alexia SJ, Boldy DP, McCaul KA. High-cost users of hospital beds in Western Australia: a population-based record linkage study. Med J Aust. 2006; 184:393–7. doi: 10.5694/j.1326-5377.2006.tb00289.x . 16618238

[10] Lee JY, Muratov S, Tarride J-E, Holbrook AM. Managing High-Cost Healthcare Users: The International Search for Effective Evidence-Supported Strategies. J Am Geriatr Soc. 2018; 66:1002–8. doi: 10.1111/jgs.15257 . 29427509

[11] Rosella LC, Kornas K, Yao Z, Manuel DG, Bornbaum C, Fransoo R, et al. Predicting High Health Care Resource Utilization in a Single-payer Public Health Care System: Development and Validation of the High Resource User Population Risk Tool. Med Care. 2018; 56:e61–e69. doi: 10.1097/MLR.0000000000000837 . 29189576 PMC6143224

[12] Frost DW, Vembu S, Wang J, Tu K, Morris Q, Abrams HB. Using the Electronic Medical Record to Identify Patients at High Risk for Frequent Emergency Department Visits and High System Costs. Am J Med. 2017; 130:601.e17-601.e22. doi: 10.1016/j.amjmed.2016.12.008 . 28065773

[13] Chechulin Y, Nazerian A, Rais S, Malikov K. Predicting Patients with High Risk of Becoming High-Cost Healthcare Users in Ontario (Canada). Healthc Policy. 2014; 9:68–79. 24726075 PMC3999564

[14] Izad Shenas SA, Raahemi B, Hossein Tekieh M, Kuziemsky C. Identifying high-cost patients using data mining techniques and a small set of non-trivial attributes. Comput Biol Med. 2014; 53:9–18. doi: 10.1016/j.compbiomed.2014.07.005 . 25105749

[15] Fleishman JA, Cohen JW. Using information on clinical conditions to predict high-cost patients. Health Serv Res. 2010; 45:532–52. doi: 10.1111/j.1475-6773.2009.01080.x . 20132341 PMC2838159

[16] Eigner I, Bodendorf F, Wickramasinghe N. Predicting high-cost patients by Machine Learning: A case study in an Australian private hospital group. EasyChair; 2019. 94–83.

[17] Moturu ST, Johnson WG, Liu H. Predictive risk modelling for forecasting high-cost patients: a real-world application using Medicaid data. IJBET. 2010; 3:114. doi: 10.1504/IJBET.2010.029654

[18] Pepe MS. Receiver Operating Characteristic Methodology. Journal of the American Statistical Association. 2000; 95:308–11. doi: 10.1080/01621459.2000.10473930

[19] Hosmer DW, Lemeshow S. Applied logistic regression. 2nd ed. New York: John Wiley; 2010.

[20] Wiens J, Shenoy ES. Machine Learning for Healthcare: On the Verge of a Major Shift in Healthcare Epidemiology. Clin Infect Dis. 2018; 66:149–53. doi: 10.1093/cid/cix731 29020316 PMC5850539

[21] Lee H-C, Yoon SB, Yang S-M, Kim WH, Ryu H-G, Jung C-W, et al. Prediction of Acute Kidney Injury after Liver Transplantation: Machine Learning Approaches vs. Logistic Regression Model. J Clin Med. 2018; 7. Epub 2018/11/08. doi: 10.3390/jcm7110428 . 30413107 PMC6262324

[22] Suzuki S, Yamashita T, Sakama T, Arita T, Yagi N, Otsuka T, et al. Comparison of risk models for mortality and cardiovascular events between machine learning and conventional logistic regression analysis. PLoS One. 2019; 14:e0221911. Epub 2019/09/09. doi: 10.1371/journal.pone.0221911 . 31499517 PMC6733605

[23] Feng J-Z, Wang Y, Peng J, Sun M-W, Zeng J, Jiang H. Comparison between logistic regression and machine learning algorithms on survival prediction of traumatic brain injuries. J Crit Care. 2019; 54:110–6. Epub 2019/08/05. doi: 10.1016/j.jcrc.2019.08.010 . 31408805

[24] Sufriyana H, Husnayain A, Chen Y-L, Kuo C-Y, Singh O, Yeh T-Y, et al. Comparison of Multivariable Logistic Regression and Other Machine Learning Algorithms for Prognostic Prediction Studies in Pregnancy Care: Systematic Review and Meta-Analysis. JMIR Med Inform. 2020; 8:e16503. Epub 2020/11/17. doi: 10.2196/16503 . 33200995 PMC7708089

[25] Boulesteix A-L, Schmid M. Machine learning versus statistical modeling. Biom J. 2014; 56:588–93. Epub 2014/02/26. doi: 10.1002/bimj.201300226 . 24615669

[26] Harris AHS, Kuo AC, Bowe TR, Manfredi L, Lalani NF, Giori NJ. Can Machine Learning Methods Produce Accurate and Easy-to-Use Preoperative Prediction Models of One-Year Improvements in Pain and Functioning After Knee Arthroplasty. J Arthroplasty. 2021; 36:112–117.e6. Epub 2020/07/20. doi: 10.1016/j.arth.2020.07.026 . 32798181

[27] Beam AL, Kohane IS. Big Data and Machine Learning in Health Care. JAMA. 2018; 319:1317–8. doi: 10.1001/jama.2017.18391 . 29532063

[28] Christodoulou E, Ma J, Collins GS, Steyerberg EW, Verbakel JY, van Calster B. A systematic review shows no performance benefit of machine learning over logistic regression for clinical prediction models. J Clin Epidemiol. 2019; 110:12–22. Epub 2019/02/11. doi: 10.1016/j.jclinepi.2019.02.004 . 30763612

[29] WHO Collaborating Centre for Drug Statistics Methodology. Guidelines for ATC classification and DDD assignment 2019. Oslo; 2018.

[30] Bundesministerium für Gesundheit. Gesetzliche Krankenversicherung. Kennzahlen und Faustformeln. 2018. Available from: https://www.bundesgesundheitsministerium.de/fileadmin/Dateien/3_Downloads/Statistiken/GKV/Kennzahlen_Daten/KF2018Bund_Juni-2018.pdf.

[31] Šimundić A-M. Measures of Diagnostic Accuracy: Basic Definitions. EJIFCC. 2009; 19:203–11. 27683318 PMC4975285

[32] Ozenne B, Subtil F, Maucort-Boulch D. The precision—recall curve overcame the optimism of the receiver operating characteristic curve in rare diseases. J Clin Epidemiol. 2015; 68:855–9. Epub 2015/02/28. doi: 10.1016/j.jclinepi.2015.02.010 . 25881487

[33] Hastie T, Tibshirani R, Friedman J. The Elements of Statistical Learning. New York, NY: Springer New York; 2009.

[34] Breiman L. Random Forest. Machine Learning. 2001; 45:5–32. doi: 10.1023/A:1010933404324

[35] Friedman JH. Greedy function approximation: A gradient boosting machine. The Annals of Statistics. 2001; 29:1189–232. doi: 10.1214/aos/1013203451

[36] Russell SJ, Norvig P. Artificial intelligence. A modern approach. Boston, Columbus, Indianapolis: Pearson; 2016.

[37] Schmidhuber J. Deep learning in neural networks: an overview. Neural Netw. 2015; 61:85–117. Epub 2014/10/13. doi: 10.1016/j.neunet.2014.09.003 . 25462637

[38] Bishop CM. Pattern recognition and machine learning. 8th ed. New York, NY: Springer; 2009.

[39] Zahedi L, Mohammadi FG, Rezapour S, Ohland MW, Amini MH. Search Algorithms for Automated Hyper-Parameter Tuning. arXiv; 2021.

[40] Liashchynskyi P, Liashchynskyi P. Grid Search, Random Search, Genetic Algorithm: A Big Comparison for NAS. arXiv; 2019.

[41] DeLong ER, DeLong DM, Clarke-Pearson DL. Comparing the Areas under Two or More Correlated Receiver Operating Characteristic Curves: A Nonparametric Approach. Biometrics. 1988; 44:837. doi: 10.2307/2531595 3203132

[42] Landry M. Machine Learning with R and H2O. Mountain View; 2018.

[43] Gedeon TD. Data mining of inputs: analysing magnitude and functional measures. Int J Neural Syst. 1997; 8:209–18. doi: 10.1142/s0129065797000227 9327276

[44] Candel A, LeDell Erin. Deep Learning with H2O. Mountain View; 2019.

[45] H2O.ai I. Variable Importance. 2021. Available from: https://docs.h2o.ai/h2o/latest-stable/h2o-docs/variable-importance.html.

[46] Heskes T, Sijben E, Bucur IG, Claassen T. Causal Shapley Values: Exploiting Causal Knowledge to Explain Individual Predictions of Complex Models. arXiv; 2020.

[47] Mangalathu S, Hwang S-H, Jeon J-S. Failure mode and effects analysis of RC members based on machine-learning-based SHapley Additive exPlanations (SHAP) approach. Engineering Structures. 2020; 219:110927. doi: 10.1016/j.engstruct.2020.110927

[48] Snider B, McBean EA, Yawney J, Gadsden SA, Patel B. Identification of Variable Importance for Predictions of Mortality From COVID-19 Using AI Models for Ontario, Canada. Front Public Health. 2021; 9:675766. Epub 2021/06/21. doi: 10.3389/fpubh.2021.675766 . 34235131 PMC8255789

[49] Wasserman L. All of Statistics. A Concise Course in Statistical Inference. New York, NY: Springer; 2004.

[50] LeDell E, Petersen M, van der Laan M. Computationally efficient confidence intervals for cross-validated area under the ROC curve estimates. Electron J Stat. 2015; 9:1583–607. doi: 10.1214/15-EJS1035 . 26279737 PMC4533123

[51] Deutsches Institut für Medizinische Dokumentation und Information (DIMDI), editor. ICD-10-GM Version 2019, Systematisches Verzeichnis, Internationale statistische Klassifikation der Krankheiten und verwandter Gesundheitsprobleme, 10. Revision, Stand: 21.September 2018. Köln: 2018 [cited 29 Jun 2019]. Available from: www.dimdi.de–Klassifikationen–Downloads–ICD-10-GM–Version2019

[52] Deutsches Institut für Medizinische Dokumentation und Information (DIMDI), editor. Anatomisch-therapeutischchemische Klassikation mit Tagesdosen. Amtliche Fassung des ATC-Index mit DDD-Angaben für Deutschland im Jahre 2019. Köln: 2019 [cited 29 Jun 2019]. Available from: https://www.dimdi.de/dynamic/de/arzneimittel/atc-klassifikation/.

[53] Luu BC, Wright AL, Haeberle HS, Karnuta JM, Schickendantz MS, Makhni EC, et al. Machine Learning Outperforms Logistic Regression Analysis to Predict Next-Season NHL Player Injury: An Analysis of 2322 Players From 2007 to 2017. Orthop J Sports Med. 2020; 8:2325967120953404. Epub 2020/09/25. doi: 10.1177/2325967120953404 . 33029545 PMC7522848

[54] Sahin EK, Colkesen I, Kavzoglu T. A comparative assessment of canonical correlation forest, random forest, rotation forest and logistic regression methods for landslide susceptibility mapping. Geocarto International. 2020; 35:341–63. doi: 10.1080/10106049.2018.1516248

[55] Muchlinski D, Siroky D, He J, Kocher M. Comparing Random Forest with Logistic Regression for Predicting Class-Imbalanced Civil War Onset Data. Polit anal. 2016; 24:87–103. doi: 10.1093/pan/mpv024

[56] Chawla NV, Japkowicz N, Kotcz A. Editorial. SIGKDD Explor Newsl. 2004; 6:1–6. doi: 10.1145/1007730.1007733

[57] H2o.ai. balance_classes; 2021. Available from: https://docs.h2o.ai/h2o/latest-stable/h2o-docs/data-science/algo-params/balance_classes.html [updated 2021; cited 2021 Nov 9].

[58] Jeni LA, Cohn JF, La Torre F de. Facing Imbalanced Data Recommendations for the Use of Performance Metrics. Int Conf Affect Comput Intell Interact Workshops. 2013; 2013:245–51. doi: 10.1109/ACII.2013.47 . 25574450 PMC4285355

[59] Prilleltensky I. Promoting well-being: time for a paradigm shift in health and human services1. Scand J Public Health Suppl. 2005; 66:53–60. doi: 10.1080/14034950510033381 . 16214724

[60] Xiao Z, Xu X, Xing H, Song F, Wang X, Zhao B. A federated learning system with enhanced feature extraction for human activity recognition. Knowledge-Based Systems. 2021; 229:107338. doi: 10.1016/j.knosys.2021.107338

[61] Xiao Z, Xu X, Xing H, Luo S, Dai P, Zhan D. RTFN: A robust temporal feature network for time series classification. Information Sciences. 2021; 571:65–86. doi: 10.1016/j.ins.2021.04.053

