# Supplementary material for: The application of machine learning to predict high-cost patients: A performance-comparison of different models using healthcare claims data
Source: PLoS One. 2023 Jan 18;18(1):e0279540. doi: 10.1371/journal.pone.0279540 (PMC9847900; doi:10.1371/journal.pone.0279540)
Supplement: S1 Table — (DOCX) [file pone.0279540.s001.docx]

**Supporting information**

**S1 Table.** Overview of all used input variables in the training/validation (2016) test (2017) dataset.

|  | **Year** | | | |
| --- | --- | --- | --- | --- |
|  | **2016 (n=20,984)** | | **2017 (n=21,148)** | |
| **Variable** | **Mean** | **SD** | **Mean** | **SD** |
| **ATC_A01** | 0.042 | 0.202 | 0.042 | 0.201 |
| **ATC_A02** | 0.264 | 0.441 | 0.261 | 0.439 |
| **ATC_A03** | 0.063 | 0.243 | 0.055 | 0.228 |
| **ATC_A04** | 0.033 | 0.178 | 0.029 | 0.167 |
| **ATC_A05** | 0.001 | 0.038 | 0.002 | 0.040 |
| **ATC_A06** | 0.025 | 0.155 | 0.025 | 0.156 |
| **ATC_A07** | 0.042 | 0.200 | 0.039 | 0.193 |
| **ATC_A08** | 0.000 | 0.000 | 0.000 | 0.000 |
| **ATC_A09** | 0.003 | 0.054 | 0.003 | 0.052 |
| **ATC_A10** | 0.119 | 0.324 | 0.121 | 0.326 |
| **ATC_A11** | 0.101 | 0.301 | 0.094 | 0.292 |
| **ATC_A12** | 0.012 | 0.109 | 0.011 | 0.102 |
| **ATC_A13** | 0.000 | 0.007 | 0.000 | 0.007 |
| **ATC_A14** | 0.000 | 0.000 | 0.000 | 0.000 |
| **ATC_A15** | 0.000 | 0.000 | 0.000 | 0.000 |
| **ATC_A16** | 0.000 | 0.010 | 0.000 | 0.010 |
| **ATC_A** | 0.476 | 0.499 | 0.469 | 0.499 |
| **ATC_B01** | 0.122 | 0.328 | 0.124 | 0.330 |
| **ATC_B02** | 0.000 | 0.021 | 0.001 | 0.028 |
| **ATC_B03** | 0.056 | 0.231 | 0.060 | 0.238 |
| **ATC_B05** | 0.011 | 0.103 | 0.012 | 0.109 |
| **ATC_B06** | 0.000 | 0.000 | 0.000 | 0.000 |
| **ATC_B** | 0.173 | 0.378 | 0.179 | 0.383 |
| **ATC_C01** | 0.039 | 0.194 | 0.039 | 0.193 |
| **ATC_C02** | 0.018 | 0.132 | 0.018 | 0.131 |
| **ATC_C03** | 0.107 | 0.309 | 0.104 | 0.306 |
| **ATC_C04** | 0.001 | 0.035 | 0.001 | 0.030 |
| **ATC_C05** | 0.018 | 0.132 | 0.017 | 0.129 |
| **ATC_C06** | 0.000 | 0.000 | 0.000 | 0.000 |
| **ATC_C07** | 0.185 | 0.388 | 0.185 | 0.388 |
| **ATC_C08** | 0.112 | 0.315 | 0.117 | 0.322 |
| **ATC_C09** | 0.251 | 0.434 | 0.253 | 0.435 |
| **ATC_C10** | 0.166 | 0.372 | 0.169 | 0.375 |
| **ATC_C** | 0.378 | 0.485 | 0.379 | 0.485 |
| **ATC_D01** | 0.064 | 0.245 | 0.063 | 0.243 |
| **ATC_D02** | 0.012 | 0.111 | 0.012 | 0.109 |
| **ATC_D03** | 0.010 | 0.099 | 0.009 | 0.097 |
| **ATC_D04** | 0.013 | 0.115 | 0.012 | 0.111 |
| **ATC_D05** | 0.006 | 0.077 | 0.005 | 0.071 |
| **ATC_D06** | 0.029 | 0.169 | 0.028 | 0.164 |
| **ATC_D07** | 0.121 | 0.326 | 0.113 | 0.317 |
| **ATC_D08** | 0.008 | 0.088 | 0.007 | 0.086 |
| **ATC_D09** | 0.002 | 0.050 | 0.003 | 0.054 |
| **ATC_D10** | 0.019 | 0.135 | 0.017 | 0.128 |
| **ATC_D11** | 0.024 | 0.152 | 0.022 | 0.147 |
| **ATC_D** | 0.240 | 0.427 | 0.230 | 0.421 |
| **ATC_G01** | 0.015 | 0.121 | 0.016 | 0.125 |
| **ATC_G02** | 0.002 | 0.043 | 0.002 | 0.041 |
| **ATC_G03** | 0.050 | 0.217 | 0.050 | 0.219 |
| **ATC_G04** | 0.056 | 0.231 | 0.061 | 0.239 |
| **ATC_G** | 0.116 | 0.320 | 0.120 | 0.325 |
| **ATC_H01** | 0.002 | 0.042 | 0.002 | 0.043 |
| **ATC_H02** | 0.083 | 0.275 | 0.083 | 0.276 |
| **ATC_H03** | 0.118 | 0.323 | 0.119 | 0.324 |
| **ATC_H04** | 0.001 | 0.033 | 0.001 | 0.036 |
| **ATC_H05** | 0.001 | 0.025 | 0.001 | 0.023 |
| **ATC_H** | 0.190 | 0.392 | 0.191 | 0.393 |
| **ATC_J01** | 0.436 | 0.496 | 0.423 | 0.494 |
| **ATC_J02** | 0.007 | 0.081 | 0.007 | 0.083 |
| **ATC_J04** | 0.001 | 0.034 | 0.001 | 0.037 |
| **ATC_J05** | 0.016 | 0.125 | 0.017 | 0.128 |
| **ATC_J06** | 0.003 | 0.054 | 0.003 | 0.055 |
| **ATC_J07** | 0.004 | 0.066 | 0.006 | 0.075 |
| **ATC_J** | 0.449 | 0.497 | 0.440 | 0.496 |
| **ATC_L01** | 0.003 | 0.051 | 0.003 | 0.050 |
| **ATC_L02** | 0.006 | 0.080 | 0.007 | 0.082 |
| **ATC_L03** | 0.004 | 0.062 | 0.003 | 0.058 |
| **ATC_L04** | 0.009 | 0.093 | 0.010 | 0.100 |
| **ATC_L** | 0.021 | 0.143 | 0.022 | 0.147 |
| **ATC_M01** | 0.439 | 0.496 | 0.428 | 0.495 |
| **ATC_M02** | 0.003 | 0.054 | 0.002 | 0.043 |
| **ATC_M03** | 0.021 | 0.142 | 0.024 | 0.154 |
| **ATC_M04** | 0.037 | 0.189 | 0.036 | 0.187 |
| **ATC_M05** | 0.014 | 0.118 | 0.013 | 0.114 |
| **ATC_M09** | 0.002 | 0.040 | 0.002 | 0.043 |
| **ATC_M** | 0.474 | 0.499 | 0.464 | 0.499 |
| **ATC_N01** | 0.004 | 0.063 | 0.004 | 0.062 |
| **ATC_N02** | 0.330 | 0.470 | 0.334 | 0.472 |
| **ATC_N03** | 0.042 | 0.201 | 0.042 | 0.200 |
| **ATC_N04** | 0.010 | 0.100 | 0.012 | 0.108 |
| **ATC_N05** | 0.094 | 0.292 | 0.096 | 0.295 |
| **ATC_N06** | 0.139 | 0.346 | 0.137 | 0.344 |
| **ATC_N07** | 0.010 | 0.101 | 0.009 | 0.096 |
| **ATC_N** | 0.449 | 0.497 | 0.450 | 0.498 |
| **ATC_P01** | 0.012 | 0.111 | 0.013 | 0.115 |
| **ATC_P02** | 0.008 | 0.086 | 0.012 | 0.107 |
| **ATC_P03** | 0.013 | 0.114 | 0.021 | 0.144 |
| **ATC_P** | 0.032 | 0.176 | 0.042 | 0.201 |
| **ATC_R01** | 0.174 | 0.379 | 0.151 | 0.358 |
| **ATC_R02** | 0.008 | 0.090 | 0.007 | 0.085 |
| **ATC_R03** | 0.156 | 0.363 | 0.156 | 0.363 |
| **ATC_R04** | 0.005 | 0.067 | 0.003 | 0.059 |
| **ATC_R05** | 0.168 | 0.374 | 0.152 | 0.359 |
| **ATC_R06** | 0.043 | 0.203 | 0.038 | 0.191 |
| **ATC_R07** | 0.000 | 0.018 | 0.000 | 0.014 |
| **ATC_R** | 0.362 | 0.480 | 0.342 | 0.474 |
| **ATC_S01** | 0.130 | 0.336 | 0.124 | 0.329 |
| **ATC_S02** | 0.022 | 0.146 | 0.020 | 0.139 |
| **ATC_S03** | 0.004 | 0.060 | 0.003 | 0.055 |
| **ATC_S** | 0.150 | 0.357 | 0.142 | 0.349 |
| **ATC_V01** | 0.006 | 0.075 | 0.006 | 0.078 |
| **ATC_V03** | 0.003 | 0.057 | 0.003 | 0.054 |
| **ATC_V04** | 0.057 | 0.231 | 0.057 | 0.231 |
| **ATC_V06** | 0.000 | 0.000 | 0.000 | 0.000 |
| **ATC_V07** | 0.000 | 0.018 | 0.001 | 0.023 |
| **ATC_V08** | 0.000 | 0.010 | 0.000 | 0.012 |
| **ATC_V09** | 0.000 | 0.000 | 0.000 | 0.000 |
| **ATC_V10** | 0.000 | 0.000 | 0.000 | 0.007 |
| **ATC_V** | 0.065 | 0.247 | 0.066 | 0.248 |
| **A00_inpatient** | 0.005 | 0.069 | 0.004 | 0.066 |
| **A15_inpatient** | 0.000 | 0.018 | 0.000 | 0.017 |
| **A20_inpatient** | 0.000 | 0.000 | 0.000 | 0.000 |
| **A30_inpatient** | 0.003 | 0.056 | 0.003 | 0.055 |
| **A50_inpatient** | 0.000 | 0.018 | 0.000 | 0.007 |
| **A65_inpatient** | 0.000 | 0.000 | 0.000 | 0.007 |
| **A70_inpatient** | 0.000 | 0.000 | 0.000 | 0.000 |
| **A75_inpatient** | 0.000 | 0.000 | 0.000 | 0.000 |
| **A80_inpatient** | 0.000 | 0.000 | 0.000 | 0.007 |
| **A90_inpatient** | 0.000 | 0.000 | 0.000 | 0.000 |
| **A92_inpatient** | 0.000 | 0.000 | 0.000 | 0.000 |
| **A_inpatient** | 0.008 | 0.092 | 0.008 | 0.087 |
| **B00_inpatient** | 0.001 | 0.032 | 0.001 | 0.029 |
| **B15_inpatient** | 0.000 | 0.021 | 0.000 | 0.022 |
| **B20_inpatient** | 0.001 | 0.032 | 0.001 | 0.034 |
| **B25_inpatient** | 0.001 | 0.025 | 0.001 | 0.027 |
| **B35_inpatient** | 0.000 | 0.015 | 0.000 | 0.017 |
| **B50_inpatient** | 0.000 | 0.014 | 0.000 | 0.007 |
| **B65_inpatient** | 0.000 | 0.007 | 0.000 | 0.000 |
| **B85_inpatient** | 0.000 | 0.000 | 0.001 | 0.025 |
| **B90_inpatient** | 0.000 | 0.007 | 0.000 | 0.000 |
| **B95_inpatient** | 0.000 | 0.000 | 0.000 | 0.000 |
| **B99_inpatient** | 0.002 | 0.043 | 0.001 | 0.038 |
| **B_inpatient** | 0.005 | 0.073 | 0.005 | 0.072 |
| **C00_inpatient** | 0.001 | 0.023 | 0.000 | 0.019 |
| **C15_inpatient** | 0.002 | 0.041 | 0.002 | 0.042 |
| **C30_inpatient** | 0.002 | 0.041 | 0.002 | 0.046 |
| **C40_inpatient** | 0.000 | 0.000 | 0.000 | 0.007 |
| **C43_inpatient** | 0.001 | 0.029 | 0.001 | 0.031 |
| **C45_inpatient** | 0.000 | 0.007 | 0.000 | 0.010 |
| **C50_inpatient** | 0.001 | 0.029 | 0.001 | 0.036 |
| **C51_inpatient** | 0.001 | 0.028 | 0.000 | 0.022 |
| **C60_inpatient** | 0.001 | 0.025 | 0.001 | 0.029 |
| **C64_inpatient** | 0.001 | 0.031 | 0.001 | 0.035 |
| **C69_inpatient** | 0.000 | 0.010 | 0.000 | 0.012 |
| **C73_inpatient** | 0.000 | 0.021 | 0.000 | 0.019 |
| **C76_inpatient** | 0.002 | 0.040 | 0.001 | 0.035 |
| **C81_inpatient** | 0.001 | 0.027 | 0.001 | 0.031 |
| **C97_inpatient** | 0.000 | 0.000 | 0.000 | 0.000 |
| **C_inpatient** | 0.009 | 0.096 | 0.011 | 0.102 |
| **D00_inpatient** | 0.000 | 0.014 | 0.000 | 0.021 |
| **D10_inpatient** | 0.005 | 0.071 | 0.004 | 0.065 |
| **D37_inpatient** | 0.006 | 0.077 | 0.004 | 0.063 |
| **D50_inpatient** | 0.002 | 0.041 | 0.002 | 0.041 |
| **D55_inpatient** | 0.001 | 0.023 | 0.000 | 0.014 |
| **D60_inpatient** | 0.002 | 0.040 | 0.001 | 0.038 |
| **D65_inpatient** | 0.001 | 0.024 | 0.001 | 0.027 |
| **D70_inpatient** | 0.000 | 0.017 | 0.000 | 0.010 |
| **D80_inpatient** | 0.000 | 0.018 | 0.000 | 0.019 |
| **D_inpatient** | 0.014 | 0.119 | 0.012 | 0.109 |
| **E00_inpatient** | 0.002 | 0.047 | 0.002 | 0.046 |
| **E10_inpatient** | 0.007 | 0.081 | 0.006 | 0.075 |
| **E15_inpatient** | 0.000 | 0.015 | 0.000 | 0.017 |
| **E20_inpatient** | 0.000 | 0.018 | 0.000 | 0.017 |
| **E40_inpatient** | 0.000 | 0.014 | 0.000 | 0.014 |
| **E50_inpatient** | 0.000 | 0.010 | 0.000 | 0.019 |
| **E65_inpatient** | 0.004 | 0.060 | 0.004 | 0.060 |
| **E70_inpatient** | 0.005 | 0.073 | 0.004 | 0.066 |
| **E_inpatient** | 0.017 | 0.128 | 0.015 | 0.122 |
| **F00_inpatient** | 0.001 | 0.037 | 0.001 | 0.035 |
| **F10_inpatient** | 0.005 | 0.074 | 0.005 | 0.069 |
| **F20_inpatient** | 0.002 | 0.044 | 0.003 | 0.051 |
| **F30_inpatient** | 0.006 | 0.079 | 0.007 | 0.085 |
| **F40_inpatient** | 0.005 | 0.068 | 0.004 | 0.066 |
| **F50_inpatient** | 0.000 | 0.022 | 0.001 | 0.023 |
| **F60_inpatient** | 0.001 | 0.038 | 0.001 | 0.034 |
| **F70_inpatient** | 0.000 | 0.010 | 0.000 | 0.007 |
| **F80_inpatient** | 0.001 | 0.032 | 0.001 | 0.035 |
| **F90_inpatient** | 0.001 | 0.025 | 0.001 | 0.027 |
| **F99_inpatient** | 0.000 | 0.007 | 0.000 | 0.007 |
| **F_inpatient** | 0.019 | 0.137 | 0.019 | 0.137 |
| **G00_inpatient** | 0.001 | 0.023 | 0.000 | 0.021 |
| **G10_inpatient** | 0.000 | 0.010 | 0.000 | 0.007 |
| **G20_inpatient** | 0.001 | 0.023 | 0.001 | 0.028 |
| **G30_inpatient** | 0.001 | 0.024 | 0.001 | 0.025 |
| **G35_inpatient** | 0.001 | 0.025 | 0.001 | 0.027 |
| **G40_inpatient** | 0.007 | 0.086 | 0.006 | 0.078 |
| **G50_inpatient** | 0.002 | 0.047 | 0.002 | 0.048 |
| **G60_inpatient** | 0.000 | 0.022 | 0.001 | 0.025 |
| **G70_inpatient** | 0.000 | 0.014 | 0.000 | 0.007 |
| **G80_inpatient** | 0.001 | 0.034 | 0.001 | 0.037 |
| **G90_inpatient** | 0.001 | 0.025 | 0.001 | 0.029 |
| **G_inpatient** | 0.014 | 0.117 | 0.013 | 0.114 |
| **H00_inpatient** | 0.001 | 0.032 | 0.001 | 0.037 |
| **H10_inpatient** | 0.000 | 0.020 | 0.000 | 0.017 |
| **H15_inpatient** | 0.001 | 0.023 | 0.001 | 0.031 |
| **H25_inpatient** | 0.001 | 0.038 | 0.002 | 0.040 |
| **H30_inpatient** | 0.002 | 0.041 | 0.001 | 0.036 |
| **H40_inpatient** | 0.001 | 0.034 | 0.002 | 0.046 |
| **H43_inpatient** | 0.000 | 0.022 | 0.000 | 0.017 |
| **H46_inpatient** | 0.000 | 0.014 | 0.000 | 0.019 |
| **H49_inpatient** | 0.001 | 0.037 | 0.001 | 0.032 |
| **H53_inpatient** | 0.002 | 0.046 | 0.001 | 0.031 |
| **H55_inpatient** | 0.000 | 0.014 | 0.000 | 0.017 |
| **H60_inpatient** | 0.000 | 0.021 | 0.000 | 0.022 |
| **H65_inpatient** | 0.003 | 0.054 | 0.003 | 0.051 |
| **H80_inpatient** | 0.002 | 0.043 | 0.001 | 0.037 |
| **H90_inpatient** | 0.001 | 0.037 | 0.002 | 0.040 |
| **H_inpatient** | 0.016 | 0.124 | 0.015 | 0.122 |
| **I00_inpatient** | 0.000 | 0.000 | 0.000 | 0.000 |
| **I05_inpatient** | 0.000 | 0.010 | 0.000 | 0.010 |
| **I10_inpatient** | 0.009 | 0.094 | 0.009 | 0.092 |
| **I20_inpatient** | 0.012 | 0.110 | 0.011 | 0.106 |
| **I26_inpatient** | 0.001 | 0.034 | 0.001 | 0.031 |
| **I30_inpatient** | 0.013 | 0.112 | 0.013 | 0.114 |
| **I60_inpatient** | 0.006 | 0.077 | 0.006 | 0.074 |
| **I70_inpatient** | 0.009 | 0.093 | 0.007 | 0.082 |
| **I80_inpatient** | 0.005 | 0.069 | 0.005 | 0.070 |
| **I95_inpatient** | 0.001 | 0.032 | 0.001 | 0.023 |
| **I_inpatient** | 0.046 | 0.209 | 0.044 | 0.205 |
| **J00_inpatient** | 0.003 | 0.053 | 0.002 | 0.049 |
| **J09_inpatient** | 0.006 | 0.080 | 0.006 | 0.077 |
| **J20_inpatient** | 0.004 | 0.062 | 0.003 | 0.053 |
| **J30_inpatient** | 0.008 | 0.088 | 0.008 | 0.087 |
| **J40_inpatient** | 0.007 | 0.083 | 0.007 | 0.083 |
| **J60_inpatient** | 0.000 | 0.022 | 0.000 | 0.017 |
| **J80_inpatient** | 0.001 | 0.025 | 0.000 | 0.022 |
| **J85_inpatient** | 0.000 | 0.015 | 0.000 | 0.014 |
| **J90_inpatient** | 0.001 | 0.028 | 0.001 | 0.023 |
| **J95_inpatient** | 0.003 | 0.057 | 0.003 | 0.057 |
| **J_inpatient** | 0.027 | 0.164 | 0.025 | 0.157 |
| **K00_inpatient** | 0.002 | 0.041 | 0.001 | 0.038 |
| **K20_inpatient** | 0.006 | 0.077 | 0.006 | 0.075 |
| **K35_inpatient** | 0.002 | 0.040 | 0.002 | 0.042 |
| **K40_inpatient** | 0.005 | 0.073 | 0.005 | 0.071 |
| **K50_inpatient** | 0.002 | 0.043 | 0.001 | 0.037 |
| **K55_inpatient** | 0.008 | 0.091 | 0.008 | 0.087 |
| **K65_inpatient** | 0.001 | 0.026 | 0.001 | 0.023 |
| **K70_inpatient** | 0.002 | 0.041 | 0.002 | 0.040 |
| **K80_inpatient** | 0.006 | 0.075 | 0.004 | 0.066 |
| **K90_inpatient** | 0.003 | 0.052 | 0.003 | 0.053 |
| **K_inpatient** | 0.031 | 0.173 | 0.029 | 0.167 |
| **L00_inpatient** | 0.004 | 0.062 | 0.005 | 0.069 |
| **L10_inpatient** | 0.000 | 0.014 | 0.000 | 0.012 |
| **L20_inpatient** | 0.002 | 0.040 | 0.001 | 0.033 |
| **L40_inpatient** | 0.001 | 0.026 | 0.001 | 0.024 |
| **L50_inpatient** | 0.001 | 0.023 | 0.000 | 0.014 |
| **L55_inpatient** | 0.000 | 0.012 | 0.000 | 0.007 |
| **L60_inpatient** | 0.001 | 0.030 | 0.001 | 0.025 |
| **L80_inpatient** | 0.002 | 0.044 | 0.001 | 0.036 |
| **L_inpatient** | 0.009 | 0.096 | 0.009 | 0.092 |
| **M00_inpatient** | 0.000 | 0.020 | 0.000 | 0.014 |
| **M05_inpatient** | 0.001 | 0.036 | 0.001 | 0.034 |
| **M15_inpatient** | 0.007 | 0.083 | 0.007 | 0.082 |
| **M20_inpatient** | 0.005 | 0.072 | 0.005 | 0.072 |
| **M30_inpatient** | 0.001 | 0.031 | 0.001 | 0.030 |
| **M40_inpatient** | 0.002 | 0.042 | 0.001 | 0.036 |
| **M45_inpatient** | 0.003 | 0.054 | 0.003 | 0.056 |
| **M50_inpatient** | 0.008 | 0.092 | 0.008 | 0.090 |
| **M60_inpatient** | 0.001 | 0.024 | 0.000 | 0.019 |
| **M65_inpatient** | 0.001 | 0.023 | 0.001 | 0.033 |
| **M70_inpatient** | 0.004 | 0.062 | 0.005 | 0.069 |
| **M80_inpatient** | 0.001 | 0.037 | 0.001 | 0.033 |
| **M86_inpatient** | 0.001 | 0.028 | 0.001 | 0.028 |
| **M91_inpatient** | 0.000 | 0.018 | 0.000 | 0.015 |
| **M95_inpatient** | 0.001 | 0.023 | 0.001 | 0.029 |
| **M_inpatient** | 0.029 | 0.168 | 0.029 | 0.169 |
| **N00_inpatient** | 0.000 | 0.014 | 0.000 | 0.014 |
| **N10_inpatient** | 0.004 | 0.065 | 0.003 | 0.055 |
| **N17_inpatient** | 0.003 | 0.056 | 0.003 | 0.055 |
| **N20_inpatient** | 0.002 | 0.050 | 0.002 | 0.048 |
| **N25_inpatient** | 0.000 | 0.014 | 0.000 | 0.010 |
| **N30_inpatient** | 0.005 | 0.069 | 0.006 | 0.074 |
| **N40_inpatient** | 0.003 | 0.053 | 0.002 | 0.046 |
| **N60_inpatient** | 0.001 | 0.029 | 0.001 | 0.027 |
| **N70_inpatient** | 0.001 | 0.028 | 0.001 | 0.026 |
| **N80_inpatient** | 0.005 | 0.068 | 0.004 | 0.061 |
| **N99_inpatient** | 0.000 | 0.015 | 0.000 | 0.012 |
| **N_inpatient** | 0.021 | 0.144 | 0.019 | 0.136 |
| **O00_inpatient** | 0.001 | 0.032 | 0.001 | 0.031 |
| **O09_inpatient** | 0.000 | 0.000 | 0.000 | 0.000 |
| **O10_inpatient** | 0.001 | 0.023 | 0.000 | 0.018 |
| **O20_inpatient** | 0.002 | 0.048 | 0.002 | 0.048 |
| **O30_inpatient** | 0.006 | 0.074 | 0.006 | 0.080 |
| **O60_inpatient** | 0.004 | 0.063 | 0.003 | 0.058 |
| **O80_inpatient** | 0.005 | 0.069 | 0.004 | 0.065 |
| **O85_inpatient** | 0.000 | 0.021 | 0.000 | 0.017 |
| **O94_inpatient** | 0.001 | 0.032 | 0.001 | 0.023 |
| **O_inpatient** | 0.012 | 0.109 | 0.012 | 0.110 |
| **P00_inpatient** | 0.000 | 0.014 | 0.000 | 0.012 |
| **P05_inpatient** | 0.001 | 0.038 | 0.002 | 0.039 |
| **P10_inpatient** | 0.000 | 0.000 | 0.000 | 0.000 |
| **P20_inpatient** | 0.000 | 0.021 | 0.001 | 0.030 |
| **P35_inpatient** | 0.000 | 0.022 | 0.000 | 0.022 |
| **P50_inpatient** | 0.001 | 0.024 | 0.000 | 0.018 |
| **P70_inpatient** | 0.000 | 0.014 | 0.000 | 0.017 |
| **P75_inpatient** | 0.000 | 0.000 | 0.000 | 0.000 |
| **P80_inpatient** | 0.000 | 0.000 | 0.000 | 0.010 |
| **P90_inpatient** | 0.000 | 0.020 | 0.000 | 0.014 |
| **P_inpatient** | 0.003 | 0.056 | 0.003 | 0.055 |
| **Q00_inpatient** | 0.000 | 0.007 | 0.000 | 0.012 |
| **Q10_inpatient** | 0.000 | 0.018 | 0.000 | 0.019 |
| **Q20_inpatient** | 0.000 | 0.020 | 0.001 | 0.024 |
| **Q30_inpatient** | 0.000 | 0.010 | 0.000 | 0.010 |
| **Q35_inpatient** | 0.000 | 0.000 | 0.000 | 0.012 |
| **Q38_inpatient** | 0.000 | 0.012 | 0.000 | 0.010 |
| **Q50_inpatient** | 0.000 | 0.018 | 0.000 | 0.021 |
| **Q60_inpatient** | 0.000 | 0.014 | 0.000 | 0.007 |
| **Q65_inpatient** | 0.001 | 0.030 | 0.001 | 0.029 |
| **Q80_inpatient** | 0.000 | 0.018 | 0.000 | 0.012 |
| **Q90_inpatient** | 0.000 | 0.000 | 0.000 | 0.007 |
| **Q_inpatient** | 0.003 | 0.050 | 0.003 | 0.052 |
| **R00_inpatient** | 0.012 | 0.107 | 0.010 | 0.102 |
| **R10_inpatient** | 0.014 | 0.116 | 0.013 | 0.113 |
| **R20_inpatient** | 0.002 | 0.043 | 0.002 | 0.041 |
| **R25_inpatient** | 0.003 | 0.051 | 0.002 | 0.048 |
| **R30_inpatient** | 0.002 | 0.044 | 0.001 | 0.037 |
| **R40_inpatient** | 0.004 | 0.063 | 0.005 | 0.068 |
| **R47_inpatient** | 0.000 | 0.022 | 0.001 | 0.028 |
| **R50_inpatient** | 0.015 | 0.123 | 0.013 | 0.113 |
| **R70_inpatient** | 0.001 | 0.032 | 0.001 | 0.031 |
| **R80_inpatient** | 0.000 | 0.007 | 0.000 | 0.000 |
| **R83_inpatient** | 0.000 | 0.015 | 0.000 | 0.010 |
| **R90_inpatient** | 0.001 | 0.025 | 0.000 | 0.012 |
| **R95_inpatient** | 0.000 | 0.000 | 0.000 | 0.000 |
| **R_inpatient** | 0.047 | 0.211 | 0.043 | 0.203 |
| **S00_inpatient** | 0.005 | 0.070 | 0.005 | 0.071 |
| **S10_inpatient** | 0.000 | 0.018 | 0.000 | 0.017 |
| **S20_inpatient** | 0.002 | 0.039 | 0.001 | 0.036 |
| **S30_inpatient** | 0.002 | 0.046 | 0.002 | 0.044 |
| **S40_inpatient** | 0.002 | 0.041 | 0.002 | 0.046 |
| **S50_inpatient** | 0.002 | 0.041 | 0.002 | 0.039 |
| **S60_inpatient** | 0.001 | 0.032 | 0.001 | 0.031 |
| **S70_inpatient** | 0.002 | 0.043 | 0.002 | 0.047 |
| **S80_inpatient** | 0.003 | 0.053 | 0.003 | 0.054 |
| **S90_inpatient** | 0.001 | 0.028 | 0.001 | 0.031 |
| **S_inpatient** | 0.016 | 0.127 | 0.017 | 0.130 |
| **T00_inpatient** | 0.000 | 0.010 | 0.000 | 0.000 |
| **T08_inpatient** | 0.001 | 0.025 | 0.001 | 0.031 |
| **T15_inpatient** | 0.000 | 0.014 | 0.000 | 0.019 |
| **T20_inpatient** | 0.000 | 0.022 | 0.000 | 0.021 |
| **T26_inpatient** | 0.000 | 0.007 | 0.000 | 0.000 |
| **T29_inpatient** | 0.000 | 0.007 | 0.000 | 0.007 |
| **T33_inpatient** | 0.000 | 0.000 | 0.000 | 0.000 |
| **T36_inpatient** | 0.001 | 0.023 | 0.000 | 0.019 |
| **T51_inpatient** | 0.000 | 0.014 | 0.000 | 0.019 |
| **T66_inpatient** | 0.001 | 0.035 | 0.001 | 0.029 |
| **T79_inpatient** | 0.000 | 0.007 | 0.000 | 0.015 |
| **T80_inpatient** | 0.005 | 0.073 | 0.004 | 0.067 |
| **T89_inpatient** | 0.000 | 0.007 | 0.000 | 0.014 |
| **T90_inpatient** | 0.000 | 0.007 | 0.000 | 0.014 |
| **T_inpatient** | 0.009 | 0.094 | 0.008 | 0.091 |
| **U00_inpatient** | 0.000 | 0.000 | 0.000 | 0.000 |
| **U50_inpatient** | 0.000 | 0.014 | 0.000 | 0.007 |
| **U55_inpatient** | 0.000 | 0.000 | 0.000 | 0.000 |
| **U60_inpatient** | 0.000 | 0.000 | 0.000 | 0.000 |
| **U69_inpatient** | 0.000 | 0.000 | 0.000 | 0.000 |
| **U80_inpatient** | 0.000 | 0.000 | 0.000 | 0.000 |
| **U99_inpatient** | 0.000 | 0.000 | 0.000 | 0.000 |
| **UUU_inpatient** | 0.000 | 0.000 | 0.000 | 0.000 |
| **U_inpatient** | 0.000 | 0.014 | 0.000 | 0.007 |
| **V01_inpatient** | 0.000 | 0.000 | 0.000 | 0.000 |
| **V_inpatient** | 0.000 | 0.000 | 0.000 | 0.000 |
| **X60_inpatient** | 0.000 | 0.000 | 0.000 | 0.000 |
| **X85_inpatient** | 0.000 | 0.000 | 0.000 | 0.000 |
| **X_inpatient** | 0.000 | 0.000 | 0.000 | 0.000 |
| **Y10_inpatient** | 0.000 | 0.000 | 0.000 | 0.000 |
| **Y35_inpatient** | 0.000 | 0.000 | 0.000 | 0.000 |
| **Y40_inpatient** | 0.000 | 0.000 | 0.000 | 0.000 |
| **Y_inpatient** | 0.000 | 0.000 | 0.000 | 0.000 |
| **Z00_inpatient** | 0.003 | 0.054 | 0.003 | 0.059 |
| **Z20_inpatient** | 0.001 | 0.030 | 0.001 | 0.023 |
| **Z30_inpatient** | 0.009 | 0.095 | 0.009 | 0.095 |
| **Z40_inpatient** | 0.002 | 0.046 | 0.002 | 0.046 |
| **Z55_inpatient** | 0.000 | 0.007 | 0.000 | 0.010 |
| **Z70_inpatient** | 0.001 | 0.029 | 0.001 | 0.024 |
| **Z80_inpatient** | 0.007 | 0.081 | 0.007 | 0.083 |
| **Z_inpatient** | 0.021 | 0.142 | 0.022 | 0.146 |
| **Age** | 42.820 | 24.866 | 42.892 | 24.816 |
| **Gender** | 0.458 | 0.498 | 0.457 | 0.498 |
| **A00_outpatient** | 0.126 | 0.331 | 0.117 | 0.322 |
| **A15_outpatient** | 0.003 | 0.054 | 0.003 | 0.052 |
| **A20_outpatient** | 0.001 | 0.032 | 0.001 | 0.035 |
| **A30_outpatient** | 0.018 | 0.134 | 0.020 | 0.140 |
| **A50_outpatient** | 0.008 | 0.086 | 0.008 | 0.090 |
| **A65_outpatient** | 0.001 | 0.029 | 0.001 | 0.029 |
| **A70_outpatient** | 0.001 | 0.025 | 0.001 | 0.028 |
| **A75_outpatient** | 0.000 | 0.000 | 0.000 | 0.007 |
| **A80_outpatient** | 0.001 | 0.025 | 0.001 | 0.026 |
| **A90_outpatient** | 0.000 | 0.000 | 0.000 | 0.000 |
| **A92_outpatient** | 0.000 | 0.000 | 0.000 | 0.007 |
| **A_outpatient** | 0.153 | 0.360 | 0.146 | 0.353 |
| **B00_outpatient** | 0.054 | 0.226 | 0.050 | 0.218 |
| **B15_outpatient** | 0.016 | 0.126 | 0.016 | 0.124 |
| **B20_outpatient** | 0.003 | 0.058 | 0.003 | 0.059 |
| **B25_outpatient** | 0.067 | 0.250 | 0.065 | 0.246 |
| **B35_outpatient** | 0.087 | 0.282 | 0.085 | 0.279 |
| **B50_outpatient** | 0.001 | 0.031 | 0.001 | 0.026 |
| **B65_outpatient** | 0.006 | 0.077 | 0.008 | 0.089 |
| **B85_outpatient** | 0.014 | 0.119 | 0.022 | 0.146 |
| **B90_outpatient** | 0.002 | 0.041 | 0.002 | 0.044 |
| **B95_outpatient** | 0.008 | 0.089 | 0.009 | 0.092 |
| **B99_outpatient** | 0.066 | 0.248 | 0.058 | 0.234 |
| **B_outpatient** | 0.265 | 0.442 | 0.262 | 0.440 |
| **C00_outpatient** | 0.001 | 0.037 | 0.002 | 0.040 |
| **C15_outpatient** | 0.024 | 0.154 | 0.024 | 0.152 |
| **C30_outpatient** | 0.004 | 0.063 | 0.004 | 0.064 |
| **C40_outpatient** | 0.000 | 0.014 | 0.000 | 0.012 |
| **C43_outpatient** | 0.012 | 0.111 | 0.013 | 0.114 |
| **C45_outpatient** | 0.001 | 0.028 | 0.001 | 0.028 |
| **C50_outpatient** | 0.010 | 0.098 | 0.010 | 0.098 |
| **C51_outpatient** | 0.004 | 0.063 | 0.004 | 0.065 |
| **C60_outpatient** | 0.008 | 0.090 | 0.008 | 0.090 |
| **C64_outpatient** | 0.006 | 0.075 | 0.006 | 0.075 |
| **C69_outpatient** | 0.001 | 0.034 | 0.001 | 0.033 |
| **C73_outpatient** | 0.002 | 0.041 | 0.002 | 0.039 |
| **C76_outpatient** | 0.007 | 0.081 | 0.008 | 0.087 |
| **C81_outpatient** | 0.006 | 0.075 | 0.006 | 0.080 |
| **C97_outpatient** | 0.000 | 0.007 | 0.000 | 0.000 |
| **C_outpatient** | 0.070 | 0.256 | 0.072 | 0.259 |
| **D00_outpatient** | 0.007 | 0.086 | 0.008 | 0.090 |
| **D10_outpatient** | 0.116 | 0.320 | 0.135 | 0.342 |
| **D37_outpatient** | 0.022 | 0.148 | 0.022 | 0.145 |
| **D50_outpatient** | 0.055 | 0.229 | 0.057 | 0.231 |
| **D55_outpatient** | 0.008 | 0.087 | 0.007 | 0.083 |
| **D60_outpatient** | 0.041 | 0.197 | 0.041 | 0.197 |
| **D65_outpatient** | 0.016 | 0.124 | 0.014 | 0.119 |
| **D70_outpatient** | 0.010 | 0.098 | 0.011 | 0.103 |
| **D80_outpatient** | 0.007 | 0.082 | 0.007 | 0.082 |
| **D_outpatient** | 0.222 | 0.415 | 0.239 | 0.427 |
| **E00_outpatient** | 0.159 | 0.366 | 0.161 | 0.368 |
| **E10_outpatient** | 0.176 | 0.381 | 0.176 | 0.381 |
| **E15_outpatient** | 0.002 | 0.041 | 0.002 | 0.041 |
| **E20_outpatient** | 0.031 | 0.173 | 0.031 | 0.172 |
| **E40_outpatient** | 0.002 | 0.040 | 0.002 | 0.041 |
| **E50_outpatient** | 0.105 | 0.307 | 0.113 | 0.316 |
| **E65_outpatient** | 0.169 | 0.374 | 0.167 | 0.373 |
| **E70_outpatient** | 0.284 | 0.451 | 0.285 | 0.452 |
| **E_outpatient** | 0.508 | 0.500 | 0.508 | 0.500 |
| **F00_outpatient** | 0.039 | 0.194 | 0.039 | 0.194 |
| **F10_outpatient** | 0.116 | 0.321 | 0.113 | 0.316 |
| **F20_outpatient** | 0.021 | 0.144 | 0.021 | 0.145 |
| **F30_outpatient** | 0.245 | 0.430 | 0.254 | 0.435 |
| **F40_outpatient** | 0.288 | 0.453 | 0.283 | 0.451 |
| **F50_outpatient** | 0.072 | 0.259 | 0.076 | 0.266 |
| **F60_outpatient** | 0.033 | 0.178 | 0.035 | 0.185 |
| **F70_outpatient** | 0.010 | 0.098 | 0.011 | 0.102 |
| **F80_outpatient** | 0.042 | 0.200 | 0.044 | 0.205 |
| **F90_outpatient** | 0.026 | 0.159 | 0.026 | 0.159 |
| **F99_outpatient** | 0.006 | 0.079 | 0.006 | 0.075 |
| **F_outpatient** | 0.521 | 0.500 | 0.523 | 0.499 |
| **G00_outpatient** | 0.001 | 0.038 | 0.002 | 0.039 |
| **G10_outpatient** | 0.001 | 0.031 | 0.001 | 0.033 |
| **G20_outpatient** | 0.021 | 0.143 | 0.021 | 0.145 |
| **G30_outpatient** | 0.009 | 0.095 | 0.010 | 0.098 |
| **G35_outpatient** | 0.003 | 0.057 | 0.003 | 0.059 |
| **G40_outpatient** | 0.168 | 0.373 | 0.171 | 0.377 |
| **G50_outpatient** | 0.064 | 0.244 | 0.066 | 0.249 |
| **G60_outpatient** | 0.068 | 0.252 | 0.068 | 0.252 |
| **G70_outpatient** | 0.002 | 0.048 | 0.002 | 0.047 |
| **G80_outpatient** | 0.015 | 0.123 | 0.015 | 0.120 |
| **G90_outpatient** | 0.015 | 0.122 | 0.018 | 0.132 |
| **G_outpatient** | 0.276 | 0.447 | 0.283 | 0.450 |
| **H00_outpatient** | 0.080 | 0.271 | 0.078 | 0.269 |
| **H10_outpatient** | 0.091 | 0.287 | 0.081 | 0.272 |
| **H15_outpatient** | 0.022 | 0.147 | 0.024 | 0.151 |
| **H25_outpatient** | 0.111 | 0.314 | 0.109 | 0.312 |
| **H30_outpatient** | 0.092 | 0.289 | 0.090 | 0.286 |
| **H40_outpatient** | 0.052 | 0.221 | 0.053 | 0.225 |
| **H43_outpatient** | 0.025 | 0.157 | 0.024 | 0.152 |
| **H46_outpatient** | 0.020 | 0.141 | 0.020 | 0.141 |
| **H49_outpatient** | 0.241 | 0.428 | 0.240 | 0.427 |
| **H53_outpatient** | 0.063 | 0.243 | 0.062 | 0.242 |
| **H55_outpatient** | 0.012 | 0.110 | 0.011 | 0.106 |
| **H60_outpatient** | 0.107 | 0.309 | 0.107 | 0.309 |
| **H65_outpatient** | 0.076 | 0.265 | 0.071 | 0.257 |
| **H80_outpatient** | 0.018 | 0.131 | 0.019 | 0.136 |
| **H90_outpatient** | 0.098 | 0.297 | 0.097 | 0.296 |
| **H_outpatient** | 0.457 | 0.498 | 0.455 | 0.498 |
| **I00_outpatient** | 0.001 | 0.023 | 0.001 | 0.028 |
| **I05_outpatient** | 0.008 | 0.091 | 0.009 | 0.095 |
| **I10_outpatient** | 0.353 | 0.478 | 0.353 | 0.478 |
| **I20_outpatient** | 0.096 | 0.295 | 0.096 | 0.295 |
| **I26_outpatient** | 0.006 | 0.080 | 0.007 | 0.084 |
| **I30_outpatient** | 0.120 | 0.324 | 0.120 | 0.325 |
| **I60_outpatient** | 0.059 | 0.236 | 0.062 | 0.240 |
| **I70_outpatient** | 0.061 | 0.239 | 0.059 | 0.236 |
| **I80_outpatient** | 0.117 | 0.322 | 0.106 | 0.308 |
| **I95_outpatient** | 0.040 | 0.195 | 0.034 | 0.182 |
| **I_outpatient** | 0.455 | 0.498 | 0.449 | 0.497 |
| **J00_outpatient** | 0.398 | 0.490 | 0.387 | 0.487 |
| **J09_outpatient** | 0.030 | 0.171 | 0.029 | 0.168 |
| **J20_outpatient** | 0.106 | 0.308 | 0.104 | 0.305 |
| **J30_outpatient** | 0.202 | 0.402 | 0.200 | 0.400 |
| **J40_outpatient** | 0.239 | 0.427 | 0.234 | 0.423 |
| **J60_outpatient** | 0.003 | 0.052 | 0.002 | 0.048 |
| **J80_outpatient** | 0.003 | 0.053 | 0.002 | 0.049 |
| **J85_outpatient** | 0.001 | 0.023 | 0.000 | 0.021 |
| **J90_outpatient** | 0.004 | 0.060 | 0.004 | 0.060 |
| **J95_outpatient** | 0.061 | 0.240 | 0.063 | 0.243 |
| **J_outpatient** | 0.630 | 0.483 | 0.621 | 0.485 |
| **K00_outpatient** | 0.050 | 0.218 | 0.054 | 0.225 |
| **K20_outpatient** | 0.199 | 0.399 | 0.197 | 0.398 |
| **K35_outpatient** | 0.003 | 0.054 | 0.003 | 0.056 |
| **K40_outpatient** | 0.032 | 0.176 | 0.032 | 0.175 |
| **K50_outpatient** | 0.055 | 0.228 | 0.051 | 0.219 |
| **K55_outpatient** | 0.129 | 0.335 | 0.128 | 0.334 |
| **K65_outpatient** | 0.003 | 0.050 | 0.003 | 0.052 |
| **K70_outpatient** | 0.070 | 0.255 | 0.070 | 0.256 |
| **K80_outpatient** | 0.041 | 0.199 | 0.040 | 0.195 |
| **K90_outpatient** | 0.012 | 0.109 | 0.012 | 0.107 |
| **K_outpatient** | 0.418 | 0.493 | 0.413 | 0.492 |
| **L00_outpatient** | 0.045 | 0.207 | 0.043 | 0.203 |
| **L10_outpatient** | 0.001 | 0.026 | 0.001 | 0.031 |
| **L20_outpatient** | 0.177 | 0.382 | 0.174 | 0.379 |
| **L40_outpatient** | 0.030 | 0.169 | 0.029 | 0.167 |
| **L50_outpatient** | 0.018 | 0.133 | 0.018 | 0.131 |
| **L55_outpatient** | 0.013 | 0.114 | 0.014 | 0.118 |
| **L60_outpatient** | 0.093 | 0.290 | 0.089 | 0.284 |
| **L80_outpatient** | 0.076 | 0.264 | 0.073 | 0.260 |
| **L_outpatient** | 0.332 | 0.471 | 0.327 | 0.469 |
| **M00_outpatient** | 0.001 | 0.030 | 0.001 | 0.030 |
| **M05_outpatient** | 0.047 | 0.211 | 0.047 | 0.212 |
| **M15_outpatient** | 0.148 | 0.355 | 0.148 | 0.355 |
| **M20_outpatient** | 0.163 | 0.370 | 0.164 | 0.370 |
| **M30_outpatient** | 0.014 | 0.116 | 0.013 | 0.114 |
| **M40_outpatient** | 0.088 | 0.283 | 0.082 | 0.275 |
| **M45_outpatient** | 0.164 | 0.371 | 0.164 | 0.371 |
| **M50_outpatient** | 0.362 | 0.480 | 0.363 | 0.481 |
| **M60_outpatient** | 0.049 | 0.215 | 0.051 | 0.221 |
| **M65_outpatient** | 0.025 | 0.156 | 0.024 | 0.153 |
| **M70_outpatient** | 0.200 | 0.400 | 0.194 | 0.395 |
| **M80_outpatient** | 0.051 | 0.220 | 0.050 | 0.218 |
| **M86_outpatient** | 0.006 | 0.080 | 0.007 | 0.081 |
| **M91_outpatient** | 0.021 | 0.144 | 0.020 | 0.139 |
| **M95_outpatient** | 0.105 | 0.306 | 0.101 | 0.302 |
| **M_outpatient** | 0.562 | 0.496 | 0.561 | 0.496 |
| **N00_outpatient** | 0.039 | 0.194 | 0.040 | 0.195 |
| **N10_outpatient** | 0.009 | 0.096 | 0.009 | 0.094 |
| **N17_outpatient** | 0.054 | 0.226 | 0.056 | 0.229 |
| **N20_outpatient** | 0.021 | 0.145 | 0.020 | 0.141 |
| **N25_outpatient** | 0.023 | 0.150 | 0.022 | 0.147 |
| **N30_outpatient** | 0.118 | 0.322 | 0.122 | 0.327 |
| **N40_outpatient** | 0.082 | 0.275 | 0.083 | 0.276 |
| **N60_outpatient** | 0.019 | 0.137 | 0.020 | 0.141 |
| **N70_outpatient** | 0.046 | 0.208 | 0.042 | 0.201 |
| **N80_outpatient** | 0.191 | 0.393 | 0.188 | 0.391 |
| **N99_outpatient** | 0.001 | 0.035 | 0.001 | 0.033 |
| **N_outpatient** | 0.394 | 0.489 | 0.394 | 0.489 |
| **O00_outpatient** | 0.007 | 0.085 | 0.005 | 0.073 |
| **O09_outpatient** | 0.019 | 0.136 | 0.018 | 0.133 |
| **O10_outpatient** | 0.001 | 0.034 | 0.001 | 0.038 |
| **O20_outpatient** | 0.017 | 0.128 | 0.017 | 0.128 |
| **O30_outpatient** | 0.009 | 0.093 | 0.009 | 0.092 |
| **O60_outpatient** | 0.004 | 0.061 | 0.003 | 0.056 |
| **O80_outpatient** | 0.002 | 0.045 | 0.002 | 0.040 |
| **O85_outpatient** | 0.003 | 0.056 | 0.003 | 0.053 |
| **O94_outpatient** | 0.005 | 0.069 | 0.005 | 0.073 |
| **O_outpatient** | 0.027 | 0.163 | 0.026 | 0.160 |
| **P00_outpatient** | 0.000 | 0.012 | 0.000 | 0.010 |
| **P05_outpatient** | 0.003 | 0.051 | 0.004 | 0.061 |
| **P10_outpatient** | 0.000 | 0.012 | 0.000 | 0.015 |
| **P20_outpatient** | 0.000 | 0.021 | 0.000 | 0.022 |
| **P35_outpatient** | 0.000 | 0.017 | 0.000 | 0.022 |
| **P50_outpatient** | 0.001 | 0.035 | 0.002 | 0.045 |
| **P70_outpatient** | 0.000 | 0.010 | 0.000 | 0.010 |
| **P75_outpatient** | 0.000 | 0.012 | 0.000 | 0.010 |
| **P80_outpatient** | 0.000 | 0.012 | 0.000 | 0.021 |
| **P90_outpatient** | 0.002 | 0.049 | 0.003 | 0.054 |
| **P_outpatient** | 0.006 | 0.079 | 0.008 | 0.092 |
| **Q00_outpatient** | 0.003 | 0.051 | 0.003 | 0.052 |
| **Q10_outpatient** | 0.004 | 0.064 | 0.004 | 0.067 |
| **Q20_outpatient** | 0.008 | 0.088 | 0.008 | 0.091 |
| **Q30_outpatient** | 0.001 | 0.023 | 0.000 | 0.017 |
| **Q35_outpatient** | 0.000 | 0.022 | 0.001 | 0.027 |
| **Q38_outpatient** | 0.003 | 0.056 | 0.003 | 0.056 |
| **Q50_outpatient** | 0.007 | 0.081 | 0.006 | 0.077 |
| **Q60_outpatient** | 0.015 | 0.121 | 0.014 | 0.117 |
| **Q65_outpatient** | 0.085 | 0.279 | 0.085 | 0.278 |
| **Q80_outpatient** | 0.013 | 0.115 | 0.012 | 0.109 |
| **Q90_outpatient** | 0.001 | 0.038 | 0.002 | 0.041 |
| **Q_outpatient** | 0.129 | 0.336 | 0.128 | 0.334 |
| **R00_outpatient** | 0.203 | 0.402 | 0.203 | 0.402 |
| **R10_outpatient** | 0.264 | 0.441 | 0.254 | 0.435 |
| **R20_outpatient** | 0.030 | 0.170 | 0.029 | 0.169 |
| **R25_outpatient** | 0.068 | 0.251 | 0.071 | 0.256 |
| **R30_outpatient** | 0.070 | 0.254 | 0.068 | 0.252 |
| **R40_outpatient** | 0.095 | 0.293 | 0.097 | 0.296 |
| **R47_outpatient** | 0.019 | 0.138 | 0.019 | 0.137 |
| **R50_outpatient** | 0.345 | 0.475 | 0.347 | 0.476 |
| **R70_outpatient** | 0.026 | 0.160 | 0.029 | 0.167 |
| **R80_outpatient** | 0.012 | 0.109 | 0.013 | 0.114 |
| **R83_outpatient** | 0.003 | 0.058 | 0.004 | 0.061 |
| **R90_outpatient** | 0.016 | 0.124 | 0.015 | 0.122 |
| **R95_outpatient** | 0.000 | 0.000 | 0.000 | 0.010 |
| **R_outpatient** | 0.636 | 0.481 | 0.635 | 0.481 |
| **S00_outpatient** | 0.031 | 0.173 | 0.030 | 0.169 |
| **S10_outpatient** | 0.007 | 0.083 | 0.006 | 0.074 |
| **S20_outpatient** | 0.013 | 0.113 | 0.012 | 0.109 |
| **S30_outpatient** | 0.012 | 0.109 | 0.011 | 0.106 |
| **S40_outpatient** | 0.012 | 0.109 | 0.013 | 0.115 |
| **S50_outpatient** | 0.011 | 0.106 | 0.012 | 0.107 |
| **S60_outpatient** | 0.026 | 0.159 | 0.026 | 0.159 |
| **S70_outpatient** | 0.008 | 0.091 | 0.008 | 0.092 |
| **S80_outpatient** | 0.027 | 0.161 | 0.028 | 0.165 |
| **S90_outpatient** | 0.032 | 0.175 | 0.030 | 0.169 |
| **S_outpatient** | 0.144 | 0.352 | 0.141 | 0.348 |
| **T00_outpatient** | 0.005 | 0.071 | 0.005 | 0.068 |
| **T08_outpatient** | 0.072 | 0.259 | 0.069 | 0.253 |
| **T15_outpatient** | 0.011 | 0.105 | 0.011 | 0.106 |
| **T20_outpatient** | 0.003 | 0.053 | 0.003 | 0.059 |
| **T26_outpatient** | 0.000 | 0.018 | 0.000 | 0.021 |
| **T29_outpatient** | 0.005 | 0.074 | 0.005 | 0.074 |
| **T33_outpatient** | 0.000 | 0.012 | 0.000 | 0.000 |
| **T36_outpatient** | 0.077 | 0.266 | 0.003 | 0.054 |
| **T51_outpatient** | 0.006 | 0.079 | 0.006 | 0.080 |
| **T66_outpatient** | 0.053 | 0.224 | 0.051 | 0.220 |
| **T79_outpatient** | 0.008 | 0.091 | 0.010 | 0.097 |
| **T80_outpatient** | 0.053 | 0.224 | 0.062 | 0.241 |
| **T89_outpatient** | 0.002 | 0.041 | 0.002 | 0.041 |
| **T90_outpatient** | 0.003 | 0.057 | 0.003 | 0.055 |
| **T_outpatient** | 0.249 | 0.432 | 0.196 | 0.397 |
| **U00_outpatient** | 0.000 | 0.000 | 0.000 | 0.000 |
| **U50_outpatient** | 0.009 | 0.092 | 0.008 | 0.087 |
| **U55_outpatient** | 0.000 | 0.000 | 0.000 | 0.000 |
| **U60_outpatient** | 0.001 | 0.036 | 0.001 | 0.035 |
| **U69_outpatient** | 0.000 | 0.007 | 0.000 | 0.018 |
| **U80_outpatient** | 0.001 | 0.033 | 0.000 | 0.022 |
| **U99_outpatient** | 0.000 | 0.000 | 0.000 | 0.000 |
| **UUU_outpatient** | 0.000 | 0.000 | 0.000 | 0.000 |
| **U_outpatient** | 0.256 | 0.436 | 0.203 | 0.402 |
| **V01_outpatient** | 0.001 | 0.029 | 0.001 | 0.026 |
| **V_outpatient** | 0.001 | 0.029 | 0.001 | 0.026 |
| **X60_outpatient** | 0.000 | 0.007 | 0.000 | 0.010 |
| **X85_outpatient** | 0.000 | 0.007 | 0.000 | 0.000 |
| **X_outpatient** | 0.000 | 0.010 | 0.000 | 0.010 |
| **Y10_outpatient** | 0.000 | 0.007 | 0.000 | 0.007 |
| **Y35_outpatient** | 0.000 | 0.000 | 0.000 | 0.000 |
| **Y40_outpatient** | 0.001 | 0.032 | 0.001 | 0.029 |
| **Y_outpatient** | 0.001 | 0.033 | 0.001 | 0.030 |
| **Z00_outpatient** | 0.470 | 0.499 | 0.491 | 0.500 |
| **Z20_outpatient** | 0.223 | 0.416 | 0.218 | 0.413 |
| **Z30_outpatient** | 0.134 | 0.340 | 0.130 | 0.337 |
| **Z40_outpatient** | 0.090 | 0.287 | 0.093 | 0.290 |
| **Z55_outpatient** | 0.023 | 0.150 | 0.023 | 0.149 |
| **Z70_outpatient** | 0.096 | 0.294 | 0.103 | 0.304 |
| **Z80_outpatient** | 0.182 | 0.386 | 0.193 | 0.394 |
| **Z_outpatient** | 0.668 | 0.471 | 0.682 | 0.466 |
| **Num_ATC_prescriptions** | 3.575 | 2.034 | 3.535 | 2.028 |
| **Num_Hospital_Diagnoses** | 0.377 | 0.893 | 0.360 | 0.837 |
| **Num_outpatient_Diagnoses** | 7.131 | 3.273 | 7.079 | 3.261 |
| **High_cost_patient_dummy** | 0.049 | 0.216 | 0.049 | 0.216 |
| **Total_costs** | 1,422.907 | 9,747.181 | 1,463.803 | 9,862.075 |
| **Need_of_Care_Duration** | 0.248 | 1.366 | 0.277 | 1.485 |
| **DMP_Duration** | 1.530 | 4.668 | 1.628 | 4.821 |
