# Supplementary material for: The application of machine learning to predict high-cost patients: A performance-comparison of different models using healthcare claims data
Source: PLoS One. 2023 Jan 18;18(1):e0279540. doi: 10.1371/journal.pone.0279540 (PMC9847900; doi:10.1371/journal.pone.0279540)
Supplement: S2 Table — (DOCX) [file pone.0279540.s002.docx]

**Supporting information**

**S2 Table.** Selected hyperparameters for all machine learning algorithms after grid search on validation data.

| **Method** | **Varied hyperparameters** | **Selected hyperparameter values** |
| --- | --- | --- |
| Artificial neural network | Number of hidden layers | 2 |
|  | Number of units per hidden layer | 10 |
|  | Activation function | Maxout with dropout |
|  | Learning rate | 0.003 |
| Random forest | Number of randomly selected features at each split | sqrt(number of variables) |
|  | Number of trees | 500 |
| Gradient boosting machine | Maximal depth of the trees | 2 |
|  | Number of trees | 500 |
