# Supplementary material for: The application of machine learning to predict high-cost patients: A performance-comparison of different models using healthcare claims data
Source: PLoS One. 2023 Jan 18;18(1):e0279540. doi: 10.1371/journal.pone.0279540 (PMC9847900; doi:10.1371/journal.pone.0279540)
Supplement: S3 Table — (DOCX) [file pone.0279540.s003.docx]

**Supporting information**

**S3 Table.** Confusion matrix of the random forest.

|  | **Predicted no HCP** | **Predicted HCP** | **Error** | **Rate** |
| --- | --- | --- | --- | --- |
| **Actual no HCP** | 16462 | 3552 | 0.18 | =3552/20014 |
| **Actual HCP1** | 249 | 883 | 0.22 | =249/1132 |
| **Totals** | 16711 | 4435 | 0.18 | =3801/21146 |
