# Supplementary material for: The application of machine learning to predict high-cost patients: A performance-comparison of different models using healthcare claims data
Source: PLoS One. 2023 Jan 18;18(1):e0279540. doi: 10.1371/journal.pone.0279540 (PMC9847900; doi:10.1371/journal.pone.0279540)
Supplement: S4 Table — (DOCX) [file pone.0279540.s004.docx]

**Supporting information**

**S4 Table.** Comparison with other studies.

| **Study** | **Num. Obs. on training data** | **Num. Obs. on test data** | **Num. of Variables** | **Method** | **AUC / c-statistics** |
| --- | --- | --- | --- | --- | --- |
| **[11]** | 58,617 | 28,721 | 12 | Logistic regression | 0.82 |
| **[15]** | 52,918 | 61,155 | - | Logistic regression | 0.857 |
| **[13]** | - | 10,300,856 | 69 | Logistic regression | 0.865 |
| **[16]** | 195,032 | - | 54 | Logistic regression | 0.976 |
| **[12]** | 21,680 | 21,431 | 11,905 | Logistic regression | 0.784 |
| **[7]** | 36,316 | 36,316 | - | Logistic regression | 0.82 |
| **[14]** | 31,704 | - | 39 | Neural network | 0.866 |
| **[6]** | - | 1,557,950 | 1059 | Penalized logistic regression | 0.836 |
| **[17]** | 40,000 | - | 437 | LogitBoost | 0.869 |
| **[7]** | 36,316 | 36,316 | - | Random forest | 0.84 |
| **[7]** | 36,316 | 36,316 | - | Gradient boosting machine | 0.84 |
| **[7]** | 36,316 | 36,316 | - | Deep neural network | 0.84 |
| **This study** | 20,984 | 21,146 | 653 | Random forest | 0.883 |
| **This study** | 20,984 | 21,146 | 653 | Gradient boosting machine | 0.878 |
| **This study** | 20,984 | 21,146 | 653 | Neural network | 0.846 |
